# Molecular mechanisms and translational advances in bladder cancer: from driver genes to precision therapy

**DOI:** 10.3389/fonc.2025.1670574

**Published:** 2025-12-08

**Authors:** Jinjie Xiang, Yuhui Luo, Baiyu Zhang, Kunbin Ke, Hao Li

**Affiliations:** Department of Urology, First Affiliated Hospital of Kunming Medical University, Kunming, China

**Keywords:** bladder cancer, molecular mechanisms, translational medicine, precision therapy, targeted therapy

## Abstract

Bladder cancer is a highly heterogeneous malignant tumor of the urinary system with high recurrence rates, posing significant challenges in its diagnosis and treatment. Advances in multi-omics research have elucidated the molecular mechanisms underlying the pathogenesis and progression of bladder cancer, including driver gene mutations (e.g., FGFR3, TP53/RB1), dysregulation of signaling pathways (such as PI3K/AKT/mTOR and RAS-MAPK), epigenetic alterations, non-coding RNA networks, tumor microenvironment remodeling, and metabolic reprogramming. This review systematically summarizes recent progress in translational research bridging molecular mechanisms to breakthroughs in precision therapy, covering the clinical applications and challenges of FGFR inhibitors, immune checkpoint inhibitors, antibody-drug conjugates, and gene therapies. Translational efforts are increasingly relying on molecular subtyping to develop subtype-specific treatment strategies. Although significant advances have been made in precision therapy for bladder cancer, critical research gaps remain, including tumor heterogeneity, therapy resistance, and insufficient validation of biomarkers. Future research directions emphasize the potential of liquid biopsy for non-invasive diagnosis and dynamic monitoring, rational combination therapies, multi-omics data integration, and artificial intelligence in advancing personalized treatment, providing a systematic and forward-looking perspective on precision medicine in bladder cancer.

## Graphical Abstract

**Figure:**
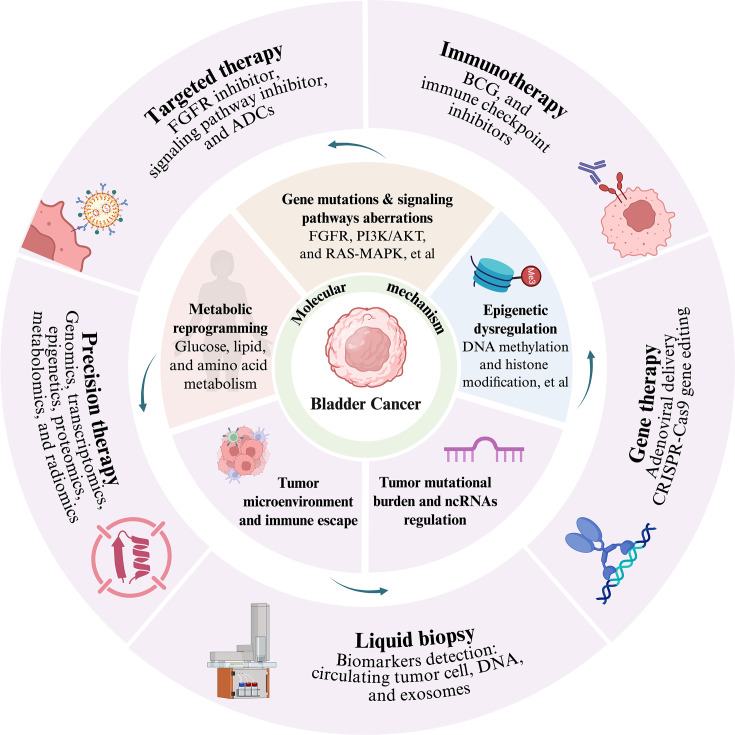
Advances in translational research on the molecular mechanisms of bladder cancer towards precision therapy: the core molecular mechanisms encompass several key dimensions: genetic mutations and aberrant signaling pathways—such as abnormalities in the FGFR, PI3K/AKT, and RAS/MAPK pathways—epigenetic dysregulation including DNA methylation and histone modifications, tumor mutational burden and non-coding RNA-mediated regulation, tumor microenvironment remodeling and immune escape, as well as metabolic reprogramming involving glucose, lipid, and amino acid metabolism. Building upon these molecular insights, multiple diagnostic and therapeutic strategies have emerged, including targeted therapy (e.g., FGFR inhibitors, signaling pathway inhibitors, and antibody-drug conjugants), immunotherapy (such as BCG and immune checkpoint inhibitors), gene therapy (e.g., adenovirus-mediated delivery and CRISPR-Cas9 gene editing), liquid biopsy via detection of biomarkers including circulating tumor cells, cell-free DNA, and exosomes, as well as precision therapy integrating multi-omics technologies spanning genomics, transcriptomics, proteomics, epigenomics, metabolomics, and radiomics.

## 1 Introduction

Globally, bladder cancer ranks as the ninth most commonly diagnosed cancer and is one of the most prevalent malignant tumors of the urinary system. Its incidence and mortality rates have been increasing worldwide. According to global cancer statistics reports, in 2022, there were approximately 614,000 new cases and 220,000 deaths from bladder cancer annually, accounting for 3.1% and 2.3% of global cancer incidence and mortality, respectively (1, 2). The biological behavior of bladder cancer is characterized by high heterogeneity and a propensity for recurrence and metastasis. Based on the tumor’s local invasion, it is classified into non-muscle-invasive bladder cancer (NMIBC) and muscle-invasive bladder cancer (MIBC), which account for approximately 80% and 20% of all bladder cancer cases, respectively (3). However, about 30%–50% of high-risk NMIBC cases progress to MIBC. This heterogeneity presents significant challenges for the diagnosis and treatment of bladder cancer. Traditional therapeutic approaches such as surgery, chemotherapy, and radiotherapy have become insufficient to meet the demands of individualized treatment. The prognosis for patients, particularly those with advanced bladder cancer, remains poor, with a 5-year survival rate of only 15%-30% (2).

In recent years, the rapid advancement of molecular biology, genomics, and proteomics technologies has led to significant progress in elucidating the molecular mechanisms of bladder cancer, providing new targets and strategies for precision therapy. From the discovery of driver genes to the dissection of signaling pathways, and from the regulation of the tumor microenvironment to the elucidation of immune evasion mechanisms, breakthroughs in these fundamental research areas are progressively translating into novel diagnostic markers and treatment modalities in clinical practice. Serving as a bridge connecting basic research with clinical applications, translational medicine has catalyzed transformative progress in the bladder cancer field, driving the leap from molecular mechanisms to precision therapy. This includes the development and application of novel therapeutic approaches such as targeted therapy, immunotherapy, and gene therapy. However, a comprehensive review that integrates recent discoveries in molecular pathogenesis with advances in translational medicine and precision therapy is still lacking. This review aims to fill this gap by synthesizing the latest evidence on molecular mechanisms, therapeutic targets, and clinical applications.

## 2 Molecular mechanisms of bladder cancer

The development and progression of bladder cancer constitute a multifactorial, multistep process involving mutations in multiple driver genes and aberrant activation of diverse signaling pathways. Field cancerization, the acquisition of pro-tumorigenic mutations and genomic alterations in normal cell lineages, has been associated with the development of bladder cancer (4). In the past decade, it has been suggested that field cancerization evolves from transformed stem cells in the urothelium that expand and drive tumor formation (‘field-first-tumor-later’ theory) (5). However, the origin of transformed cells among normal-appearing urothelial cells is unclear, with original speculation that cancer cells from tumors migrate in the urothelium or are shed from tumors and implanted between normal cells (6). This is referred to as the ‘tumor-first-field-later’ theory. Both theories may explain frequent recurrences of clonally related bladder tumors that develop years apart (7). Whole-organ mapping studies demonstrated that genetic alterations can be divided into two categories: low-frequency mutations and high-frequency mutations increasing with disease progression. Patients with a high level of field cancerization had poor survival, and tumors from these patients harboured a high mutational burden, high neoantigen load and high tumor-associated CD8^+^ T cell exhaustion (8).

In recent years, whole-genome sequencing and exome sequencing technologies have identified a series of critical driver genes in bladder cancer. Mutations or dysregulated expression of these genes contribute to various biological processes, including cell proliferation, apoptosis, differentiation, invasion, and metastasis. Importantly, non-synonymous mutations in known bladder cancer driver genes, such as chromatin remodelling genes and TP53, STAG2 and PIK3CA, have been identified in non-diseased bladders as well as in histologically tumor-free urothelium from patients with bladder cancer (8).

### 2.1 Driver gene mutations and signaling pathway aberrations

#### 2.1.1 Aberrant expression of FGFR

Mutations or amplifications in the fibroblast growth factor receptor (FGFR) gene family are among the most frequent alterations in the development and progression of bladder cancer. Studies indicate that approximately 60% of NMIBC cases harbor FGFR3 gene mutations or overexpression, compared to about 15% in MIBC (9–11). Aberrant activation of the FGFR signaling pathway promotes bladder cancer progression through multiple mechanisms. On the one hand, FGFR3 activation can induce epithelial-mesenchymal transition (EMT), enhancing cancer cell migration and invasion capabilities. On the other hand, it promotes tumor angiogenesis and immune evasion, thereby fostering a conducive microenvironment for tumor growth and metastasis (10). Activating mutations in FGFR3 (e.g., R248C, S249C, Y373C) can also constitutively activate downstream PI3K/Akt/mTOR and RAS-MAPK pathways through ligand-independent dimerization, promoting cell proliferation and suppressing apoptosis (10, 11). Research demonstrates that FGFR expression remains more stable in highly aggressive tumors, and FGFR3 overexpression or aberrant activation of other FGFR family members (e.g., FGFR1) can promote tumor progression and metastasis via ligand-dependent mechanisms (12). Patients with FGFR3-mutant bladder cancer exhibit greater sensitivity to FGFR inhibitor therapy. For instance, erdafitinib, a selective pan-FGFR inhibitor, has been approved by the U.S. Food and Drug Administration (FDA) for the treatment of locally advanced or metastatic urothelial carcinoma (UC) harboring FGFR2/3 mutations or fusions. In platinum-resistant patients with FGFR alterations, erdafitinib achieved an objective response rate (ORR) of 40%, significantly higher than the 11.5% observed with standard chemotherapy (13, 14). Furthermore, erdafitinib has demonstrated promising potential in the treatment of NMIBC, with related studies reporting encouraging outcomes. Specifically, among high-risk, BCG-unresponsive NMIBC patients (particularly those with carcinoma *in situ*) harboring FGFR3/2 alterations, erdafitinib has exhibited favorable and durable efficacy. However, it has not yet been formally approved for clinical use in NMIBC (11). These findings underscore the pivotal role of the FGFR signaling pathway in the molecular mechanisms of bladder cancer and highlight its clinical value as a target for precision therapy.

#### 2.1.2 Aberrant expression of TP53 and RB1

TP53 and RB1 are the most frequently inactivated tumor suppressor genes in MIBC. Inactivating mutations in these genes lead to dysregulation of the cell cycle and genomic instability. TP53 gene mutation represents the most common genetic alteration in MIBC, occurring in approximately 50%-60% of cases, while RB1 loss is frequently observed in the basal-like subtype (15). Studies demonstrate that TP53 mutation not only results in loss of cell cycle checkpoint function, diminished DNA damage repair capacity, and promotion of tumor initiation and progression, thereby correlating strongly with an aggressive phenotype, chemotherapy resistance, and poor prognosis in bladder cancer, but also promotes the formation of an inflammatory microenvironment via activation of the NF-κB signaling pathway. Conversely, RB1 loss drives uncontrolled cell cycle progression through E2F3 amplification (16). Concurrent TP53/RB1 double mutations are commonly found in the neuroendocrine variant (accounting for approximately 3% of cases) and are associated with a dismal prognosis (16).

Despite the high frequency of TP53 mutations in bladder cancer, developing successful single-target p53 therapies has remained challenging, partly due to the complexity of p53 regulation and functional restoration. Emerging strategies, such as small activating RNA designed to upregulate wild-type p53 expression or restore function in mutant p53, represent novel approaches beyond conventional targeted therapy. These innovative directions may open new avenues for precision intervention in p53-dysregulated bladder cancers.

#### 2.1.3 PI3K/AKT/mTOR signaling pathway

The PI3K/AKT/mTOR pathway represents one of the most frequently activated signaling networks in bladder cancer, with alterations observed in approximately 50% of cases (17). Activation of this pathway can occur through multiple mechanisms, including PIK3CA mutations, PTEN loss, or hyperphosphorylation of AKT. PIK3CA, a key driver gene, frequently harbors mutations that attenuate the inhibitory function of the p85 regulatory subunit while enhancing the membrane lipid-binding capacity of the p110α catalytic subunit, leading to constitutive PI3K activation (17). Such mutations are present in 20–30% of NMIBC cases and often co-occur with FGFR3 mutations (11). PTEN, a critical negative regulator, terminates pathway signaling by dephosphorylating PIP3 to PIP2. In bladder cancer, PTEN loss can occur through genetic mutation (10–15%), promoter methylation (15–20%), or loss of heterozygosity (30–40%). It is more prevalent in MIBC and is associated with increased tumor aggressiveness (11, 17). Elevated phosphorylation of AKT1/2 is observed in 40–50% of bladder cancer cases, independent of PIK3CA or PTEN alterations, and may result from upstream receptor tyrosine kinase (e.g., FGFR3 or EGFR) activation (10, 17). PI3K/AKT/mTOR signaling activation suppresses apoptosis and promotes cell cycle progression, metabolic reprogramming, and proliferation. It also facilitates tumor angiogenesis via upregulation of vascular endothelial growth factor (VEGF) expression (10, 18). Clinically, PIK3CA mutations are associated with significantly reduced recurrence-free survival. Patients with complete PTEN loss exhibit a 40% shorter overall survival (OS) compared to those with wild-type PTEN (11, 17). In FGFR3-mutant bladder cancers, concurrent PI3K/AKT activation predicts reduced response to FGFR inhibitors such as erdafitinib (11). In NMIBC, PI3K/AKT activation often cooperates with FGFR3 mutations, whereas in MIBC, it frequently co-occurs with TP53/RB1 inactivation (10, 11). PTEN-deficient tumors show a 30% higher resistance rate to cisplatin-based chemotherapy, while PIK3CA-mutant cases may benefit from PI3K inhibitors (17).

Gαi3, a member of the inhibitory G protein α subunit family, plays a critical role in the development and progression of bladder cancer. Studies demonstrate that Gαi3 is significantly overexpressed in bladder cancer tissues, and its expression level correlates closely with tumor grade, stage, invasiveness, and lymph node metastasis (19). Gαi3 interacts with receptor tyrosine kinases such as the epidermal growth factor receptor (EGFR) and FGFR, mediating the activation of the downstream PI3K/AKT/mTOR signaling cascade to promote the proliferation, migration, and invasion of bladder cancer cells. Research shows that Gαi3 knockout significantly inhibits the growth of bladder cancer xenograft tumors in nude mice, concomitant with reduced phosphorylation levels of AKT and S6K and increased apoptosis (19). These findings establish Gαi3 as a potential novel therapeutic target for bladder cancer, providing a theoretical foundation for developing therapeutic strategies targeting Gαi3.

#### 2.1.4 RAS-MAPK signaling pathway

Activation of the RAS-MAPK pathway serves as a central axis regulating proliferation and differentiation in bladder cancer, playing a critical role in its pathogenesis. Aberrant activation primarily arises from RAS mutations, upstream receptor-driven signaling, or dysregulation within the cascade (20). KRAS mutations represent the predominant subtype (5–15% incidence), leading to constitutive GTP-bound activation of RAS protein and impaired inactivation by GAP proteins (10). NRAS mutations are less frequent (2–5%) and are predominantly observed in basal-like MIBC (11). FGFR3 mutations activate RAS through recruitment of the GRB2–SOS complex via the adaptor protein FRS2α, while EGFR amplification or mutations trigger the pathway through similar mechanisms (10, 20). The FGFR3–TACC3 fusion protein activates RAF via constitutive dimerization, bypassing RAS-dependent steps (10). BRAF mutations (1–3%), predominantly V600E, also result in sustained RAF kinase activation. Elevated phosphorylation of MEK1/2 is observed in 20–25% of bladder cancers, independent of RAS/BRAF alterations, and is associated with tumor aggressiveness (11, 17, 20). The RAS-MAPK pathway frequently exhibits crosstalk with the PI3K/AKT pathway, collectively driving tumor progression. For instance, FGFR3 mutations activate both RAS-MAPK and PI3K/AKT signaling. Furthermore, ERK-mediated phosphorylation of TSC2 relieves its inhibition of mTORC1, thereby amplifying PI3K/AKT downstream effects. This synergistic interaction significantly enhances tumor survival advantage and therapy resistance (10, 17). Clinically, RAS-MAPK activation is more prevalent in MIBC (25–30%) than in NMIBC (10–15%), with enrichment in basal-like and neuroendocrine-like subtypes (11). Patients with KRAS mutations exhibit reduced ORR to platinum-based chemotherapy (15% vs. 40% in wild-type) and a 20% lower response rate to immune checkpoint inhibitors such as pembrolizumab (11, 20). In FGFR3-mutant bladder cancer, concurrent RAS-MAPK activation is associated with a 2.1-fold higher recurrence risk and shorter time to resistance to erdafitinib (3.5 months vs. 5.6 months in wild-type) (11). Although targeted therapies have demonstrated preliminary efficacy, overcoming resistance remains a major clinical challenge.

#### 2.1.5 Other signaling pathway

The Hippo-YAP pathway serves as a critical oncogenic signaling network in bladder cancer, centered on the Hippo kinase cascade (MAP4K, MST1/2, LATS1/2) and the nuclear transcriptional module (YAP/TAZ–TEAD). Approximately 60–70% of bladder cancer patients exhibit aberrant activation of this pathway (21–23). Activation mechanisms are multidimensional: for instance, mutations in GNA13 and overexpression of NUAK2 significantly enhance YAP/TAZ transcriptional activity, promoting malignant transformation and sustained tumor progression (24). Increased stiffness of the extracellular matrix (ECM) facilitates YAP dephosphorylation via the integrin–FAK–CDC42–PP1A axis, thereby accelerating the transition from NMIBC to MIBC (25). Clinically, YAP expression levels positively correlate with pathological grade and depth of invasion in bladder cancer (22). YAP/TAZ activation represents a key mechanism underlying resistance to chemotherapy agents such as cisplatin and docetaxel (26). In MIBC, YAP activation frequently co-occurs with molecular features like FGFR3 mutations and p53 inactivation, and is more prevalent in basal-like subtypes, suggesting its involvement in subtype-specific malignant progression (27).

Abnormalities in the PPARG signaling pathway exhibit remarkable subtype-specific characteristics in bladder cancer: luminal tumors highly express markers such as PPARG, FOXA1, and KRT20, are often classified as NMIBC, yet demonstrate high recurrence rates; whereas basal/squamous (BASQ) subtypes show low PPARG expression, are typically MIBC, and progress rapidly (28, 29). In BASQ bladder cancers, PPARG expression is significantly downregulated. In luminal subtypes, 20–25% of cases harbor PPARG gene amplification or mutations in its binding partner retinoic acid X receptor (RXR) α, leading to constitutive pathway activation (30). Core regulatory mechanisms involve multi-layered molecular interactions and microenvironmental responses. PPARG, a nuclear receptor transcription factor, functions as a heterodimer with RXR. Its activation induces differentiation of bladder basal progenitor cells into terminally differentiated superficial cells and promotes cell cycle exit via p21 upregulation, thereby suppressing tumorigenesis (28). The MEK/ERK pathway inhibits PPARG transcriptional activity and promotes its degradation through phosphorylation—a mechanism particularly active in BASQ bladder cancer and central to PPARG downregulation in this subtype. Furthermore, PPARG directly suppresses expression of core NF−κB pathway components, dampening immunoinflammatory responses. This contributes to an immune-cold microenvironment in luminal tumors, resulting in significantly lower response rates to PD-1/PD-L1 inhibitors compared to BASQ subtypes (29).

The aberrant activation of the SHH signaling pathway exhibits significant heterogeneity in bladder cancer, characterized primarily by dysregulated activation of the Gli transcription factor family (particularly Gli3) and imbalance in non-coding RNA regulation. This phenomenon occurs at a significantly higher frequency in MIBC compared to NMIBC (31, 32). Gli3 expression levels show a positive correlation with pathological grade, depth of invasion, and lymph node metastasis in bladder cancer. Patients with high Gli3 expression have significantly shorter OS and recurrence-free survival (32, 33). The abnormal activation of the SHH pathway is primarily mediated through the following mechanisms: microRNA miR-7-5p is significantly downregulated in bladder cancer, where it directly binds to the 3’ untranslated region of Gli3 to inhibit its protein expression. The loss of miR-7-5p leads to derepressed accumulation of Gli3, subsequently activating the transcription of downstream target genes and promoting cell proliferation and migration (32). The SHH pathway can also crosstalk with the PI3K/AKT pathway, whereby activated AKT promotes the degradation of negative regulators of Gli through phosphorylation, thereby enhancing Gli transcriptional activity (34). Upon nuclear translocation, activated Gli family proteins bind to Gli-binding sites in the promoter regions of target genes, regulating the expression of cell cycle genes and EMT-related genes, ultimately driving bladder cancer progression (35).

Aberrant activation of the NOTCH signaling pathway also contributes to the development and progression of bladder cancer. Mutations in NOTCH1 or overexpression of its ligands can promote the proliferation and EMT of bladder cancer cells (36).

### 2.2 Tumor mutational burden and mutational signatures

Bladder cancer, particularly MIBC, is among the malignancies with the highest mutational burden. This inherent genomic instability stems from exposure to various carcinogens (such as aromatic amines from tobacco smoking) and defects in endogenous DNA repair mechanisms. Tumor mutational burden (TMB) and specific mutational signatures have emerged as key biomarkers for understanding the mechanisms of tumorigenesis and predicting treatment response.

TMB is generally defined as the total number of somatic nonsynonymous mutations per megabase. High TMB (typically defined as ≥10 mut/Mb) is highly prevalent in bladder cancer, accounting for approximately 20%–30% of MIBC cases (37). Its causes are primarily associated with three major factors: defects in DNA repair pathways, activation of endogenous mutagenic enzymes, and exogenous stressors (38, 39). In bladder cancer, TMB profoundly influences disease progression by regulating neoantigen generation, genomic stability, and tumor-immune interactions (39, 40). Mutational signatures refer to specific patterns of somatic mutations (e.g., C>A, T>G) in the genome, reflecting specific endogenous or exogenous carcinogenic processes active during tumor development. APOBEC is the most prevalent endogenous mutational signature in bladder cancer, observed in over 70% of cases (41). It produces characteristic SBS2 (C→T) and SBS13 (C→A/G) mutational patterns and serves as a major driver of genomic evolution and heterogeneity in bladder cancer. It is also associated with high TMB and a trend toward better response to immunotherapy (39, 42). Exogenous factors increase TMB through direct DNA damage or activation of mutagenic pathways, with platinum-based chemotherapy and tobacco smoking being typical examples (39, 43). Dysfunctional DNA repair systems represent a major molecular basis for high-TMB bladder cancer, involving deficiencies in mismatch repair (MMR), DNA damage response, and polymerase proofreading (39, 40). For instance, although mutations in MMR genes are relatively rare in bladder cancer (2.2%–9.4%), they significantly elevate TMB and generate a high number of insertion/deletion mutations. Patients with such alterations show exceptional sensitivity to immune checkpoint inhibitors (ICIs) (39, 44). Due to their abundant neoantigen load, high-TMB bladder cancers exhibit an ORR of 29% to PD-(L)1 inhibitors, significantly higher than that in low-TMB groups (6%). High TMB is also associated with significantly improved OS in stage III bladder cancer, though not in stages II and IV (40, 45).

In summary, the TMB status and mutational signatures collectively shape the molecular landscape of bladder cancer: high-TMB tumors are characterized by APOBEC3-mediated early clonal mutations and DNA repair deficiencies, whereas low-TMB tumors are primarily influenced by environmental mutagenesis (39, 45). Integrated analysis of TMB, mutational signatures, molecular subtypes, and PD-L1 expression will form the basis for more refined personalized treatment strategies in the future. This approach not only provides new insights into tumor biology but, more importantly, offers a robust foundation for decision-making in precision immunotherapy and chemotherapy selection.

### 2.3 Epigenetic dysregulation

Epigenetic regulation, which modulates gene expression through mechanisms such as DNA methylation, histone modification, and chromatin remodeling, plays a central role in the initiation and progression of bladder cancer. Unlike irreversible genetic mutations, epigenetic alterations are reversible, offering promising novel strategies for the prevention and treatment of bladder cancer.

#### 2.3.1 Aberrant DNA methylation: from single genes to regulatory networks

Abnormal DNA methylation is one of the earliest confirmed epigenetic alterations in bladder cancer, characterized by an imbalance between genome-wide hypomethylation and promoter-specific hypermethylation of certain tumor suppressor genes. This imbalance persists throughout tumor progression. Promoter hypermethylation does not occur in isolated genes but rather leads to coordinated silencing of multiple genes. For instance, the key tumor suppressor gene RASSF1A exhibits promoter hypermethylation in 80% of bladder cancer cell lines. Moreover, methylation was detected in 97% of tumors with RASSF1A silencing. This abnormality is significantly associated with deeper tumor invasion, occurring more frequently in MIBC than in NMIBC, and serves as an independent marker of poor prognosis (46). Other frequently hypermethylated targets include p14ARF and APC. In a study of 113 bladder cancer cases, promoter methylation of p14ARF was detected in 38% of tumor tissues and 32% of urine samples, while APC methylation was observed in 54% of tissues and 46% of urine samples. Methylation of both genes positively correlated with higher tumor grade (p = 0.002 and p = 0.02, respectively) (47). Additionally, methylation of DAPK1 and p16INK4A is also common. DAPK1 hypermethylation promotes tumor survival by silencing apoptotic regulatory functions, whereas p16INK4A methylation accelerates proliferation through inactivation of cell cycle checkpoints. These two genes often form a methylation panel with RASSF1A and APC, enabling non-invasive diagnosis and recurrence monitoring of bladder cancer via urine testing (47). On the other hand, genome-wide hypomethylation contributes to carcinogenesis primarily by activating proto-oncogenes and compromising genomic stability. For example, hypomethylation in repetitive sequence regions can lead to chromosomal translocations and gene fusions, while hypomethylation in regulatory regions of certain proto-oncogenes (e.g., FGFR3) may cause their aberrant activation, facilitating malignant transformation of bladder cancer cells (48).

#### 2.3.2 Dysregulated histone modifications: an imbalance network centered on acetylation and methylation

Histone modifications, including acetylation, methylation, and phosphorylation, play crucial roles in epigenetic regulation. Disruption of their dynamic balance represents another key feature of epigenetic abnormality in bladder cancer, among which dysregulated acetylation mediated by histone deacetylases (HDACs) has been most extensively studied. Members of the HDAC family exhibit subtype-specific abnormalities in bladder cancer: Class I HDACs (HDAC1, 2, 3) are overexpressed in 40%–59% of bladder cancers. Nuclear localization of HDAC1/2 is associated with higher tumor grade and promotes cell cycle progression by repressing the transcription of tumor suppressor genes such as p21 and p57. Among Class II HDACs, HDAC4 shows significantly increased positivity in tumor tissues, while HDAC6 collaborates with SIRT2 (a Class III HDAC) to enhance cell migration and invasion by deacetylating cortical actin. SIRT7, another Class III HDAC, is highly expressed in high-grade tumors, and its knockdown induces apoptosis in bladder cancer cells (48). In contrast, mutations or loss of histone acetyltransferases such as CREBBP/EP300 impair transcriptional activation of tumor suppressor genes and are associated with aggressive phenotypes in MIBC. Aberrant histone methylation is equally critical. For instance, mutations in the lysine methyltransferase KMT2D, which occur frequently in bladder cancer, reduce H3K4 trimethylation (a transcriptional activation mark) and suppress tumor suppressor gene expression. Inactivation of the lysine demethylase KDM6A enhances AP-1 pathway activity, driving the transition from luminal to basal-like subtypes and increasing invasiveness (48). Together, these aberrant modifications remodel chromatin structure to form a transcriptionally permissive environment conducive to tumor proliferation.

#### 2.3.3 Epigenetic regulators: from enzymatic dysfunction to metabolite-mediated cascades

Mutations or dysregulation of epigenetic regulators themselves are major contributors to epigenetic imbalance in bladder cancer, involving DNA modifiers, histone modifiers, and metabolism-linked regulators. Although IDH1/IDH2 mutations are relatively rare in bladder cancer (∼2%), their dysfunction has notable ‘metabolite–epigenetic’ cascade effects: mutant IDH1 (e.g., R132H) loses its normal catalytic activity and instead produces high levels of 2-hydroxyglutarate (2-HG) in an NADPH-dependent manner. 2-HG competitively inhibits DNA demethylases and histone demethylases, leading to genome-wide DNA hypermethylation and disrupted histone methylation patterns, ultimately repressing tumor suppressor gene expression (49). Abnormalities in the TET–TDG pathway directly disrupt active DNA demethylation: TET2, a key enzyme catalyzing the oxidation of 5-methylcytosine to 5-hydroxymethylcytosine (hmC), is frequently downregulated in bladder cancer, resulting in reduced hmC accumulation. Meanwhile, functional defects in thymine DNA glycosylase (TDG), which is responsible for excising the oxidation products 5-formylcytosine and 5-carboxylcytosine, impair base excision repair-mediated demethylation, thereby maintaining the silent state of tumor suppressor genes. Notably, the TET–TDG pathway also regulates non-CpG sites (e.g., CA), and its dysregulation can influence the differentiation state of bladder cancer cells (50). Additionally, overexpression of DNA methyltransferase DNMT1 enhances promoter methylation efficiency, and mutations in the chromatin remodeler ARID1A disrupt chromatin accessibility, leading to aberrant gene expression. Both mechanisms are closely associated with malignant progression of bladder cancer (48).

In summary, epigenetic abnormalities in bladder cancer are not isolated events affecting single genes, but rather constitute a complex regulatory network centered on DNA methylation imbalances, dysregulated histone modifications, and dysfunctional epigenetic regulators. This network not only drives tumorigenesis but also offers potential diagnostic biomarkers and therapeutic targets.

### 2.4 Role of non-coding RNAs

Non-coding RNAs (ncRNAs) are a class of RNA molecules that do not encode proteins but possess important regulatory functions. This category includes long non-coding RNAs (lncRNAs), microRNAs (miRNAs), and circular RNAs (circRNAs), among others. Research in recent years has demonstrated that ncRNAs play critical regulatory roles in the development, progression, invasion, metastasis, and therapy resistance of bladder cancer.

LncRNAs, defined as ncRNAs exceeding 200 nucleotides in length, exert critical regulatory roles in gene expression during bladder cancer pathogenesis through diverse mechanisms, including chromatin modification, transcriptional regulation, and RNA interference. Studies have demonstrated that aberrant expression of numerous lncRNAs correlates with the initiation and progression of bladder tumors. For instance, lncRNA UCA1 is highly expressed in bladder cancer. It functions as a molecular sponge for miR-143, thereby regulating c-Myc expression and promoting tumor cell proliferation (51). Conversely, elevated expression of lncRNA GAS6-AS2 is associated with an aggressive phenotype and poor prognosis in bladder cancer. GAS6-AS2 promotes tumor cell proliferation and migration by modulating the PI3K/AKT signaling pathway (52). Furthermore, lncRNAs such as BLACAT2 and LNMAT1 have been implicated in the lymphatic metastasis of bladder cancer (53, 54). BLACAT2 promotes lymphatic metastasis by interacting with the WDR5/MLL complex to enhance histone H3 lysine 4 trimethylation (H3K4me3). This epigenetic modification upregulates vascular endothelial growth factor C (VEGF-C) expression, inducing tumor-associated lymphangiogenesis (53). LNMAT1, on the other hand, facilitates the recruitment of tumor-associated macrophages (TAMs), promoting VEGF-C secretion and establishing a pro-lymphangiogenic and pro-metastatic microenvironment (54). Another lncRNA, LNMAT2, is secreted via the exosomal pathway from bladder cancer cells. Upon uptake by lymphatic endothelial cells (LECs), LNMAT2 increases H3K4me3 levels at the PROX1 promoter region. This stimulates LEC proliferation and lymphangiogenesis, ultimately driving tumor lymphatic metastasis (55). Collectively, these studies reveal the intricate regulatory network orchestrated by lncRNAs in the pathogenesis, progression, and metastasis of bladder cancer, providing novel therapeutic perspectives for developing lncRNA-targeted strategies.

MiRNAs are small non-coding RNAs approximately 22 nucleotides in length that regulate gene expression by binding to the 3’ untranslated region (3’UTR) of target messenger RNAs (mRNAs), leading to mRNA degradation or translational repression. Functioning as crucial regulators of gene expression, miRNAs exhibit aberrant expression patterns in bladder cancer and act as tumor suppressors or oncogenes, participating in diverse biological processes such as cell proliferation, apoptosis, invasion, and metastasis. Research indicates that the miR-200 family inhibits invasion, metastasis, and cisplatin resistance in bladder cancer cells by modulating ZEB1/2 expression, thereby influencing the EMT process (56). miR-21 overexpression is frequently observed in bladder cancer. It promotes tumor angiogenesis and bladder cancer progression by targeting PTEN, which induces activation of the PI3K/AKT signaling pathway and suppresses maspin expression, ultimately leading to the upregulation of VEGF-C (57, 58). Conversely, miR-128 expression is downregulated in bladder cancer tissues. It inhibits tumor lymphangiogenesis and lymphatic metastasis by targeting VEGF-C (59). Furthermore, the aberrant expression of miR-141 and miR-200b in the urine of bladder cancer patients serves as a diagnostic biomarker for lymphatic metastasis. The diagnostic efficacy of these miRNAs has been evaluated using receiver operating characteristic (ROC) curve analysis (60). Collectively, these studies demonstrate that miRNAs participate in the initiation, progression, and metastasis of bladder cancer by regulating the expression of multiple oncogenes and tumor suppressor genes. Their potential as diagnostic biomarkers and therapeutic targets warrants further investigation.

CircRNAs are a class of non-coding RNAs characterized by a closed circular structure, exhibiting features such as high stability and strong tissue specificity. Recent research has revealed that circRNAs also play significant regulatory roles in bladder cancer. For example, circRNA_000520 demonstrates low expression in bladder cancer. It inhibits tumor cell proliferation and metastasis through the Lin28a/PTEN/PI3K signaling cascade (61).

Although the regulatory roles of ncRNAs in bladder cancer have been extensively uncovered, their clinical translation still faces several challenges. First, there is a need to deeply investigate the complex interactive networks among different ncRNAs, and how they cooperatively regulate key signaling pathways in bladder cancer. Given the high tissue specificity and stability of ncRNAs, exploring their potential as non-invasive liquid biopsy biomarkers (based on urine or blood) for early diagnosis, prognosis assessment, and prediction of lymphatic metastasis holds great promise. Second, integrating ncRNA expression profiles with molecular subtypes of bladder cancer will help reveal the distinct biological behaviors of different tumor subtypes and provide a theoretical foundation for developing personalized treatment strategies based on ncRNAs. Finally, elucidating the specific mechanisms by which ncRNAs mediate intercellular communication within the TME will be crucial for overcoming tumor heterogeneity and therapy resistance. Translating these fundamental discoveries into clinical applications ultimately holds the potential to provide novel diagnostic tools and precision therapeutic options for patients with bladder cancer.

### 2.5 Tumor microenvironment and immune escape

The tumor microenvironment (TME) constitutes the complex milieu in which tumor cells reside, encompassing immune cells, fibroblasts, vascular endothelial cells, the ECM, and various cytokines. The development and progression of bladder cancer are closely associated with TME remodeling. Alterations in the TME not only provide favorable conditions for tumor cell growth and metastasis but also contribute to tumor immune evasion.

The infiltration patterns and functional states of immune cells within the TME are intimately linked to the pathogenesis, progression, and prognosis of bladder cancer. Among these immune cells, TAMs are key participants. TAMs exhibit dual roles within the bladder tumor microenvironment. M1-polarized macrophages possess anti-tumor functions, whereas M2-polarized macrophages promote tumor angiogenesis, EMT, and immune suppression by secreting anti-inflammatory factors and pro-angiogenic factors such as TGF-β, IL-10, and VEGF-C (62, 63). Furthermore, bladder cancer-derived CCL2 recruits TAMs to the tumor microenvironment. In turn, TAM-secreted VEGF-C further promotes lymphangiogenesis, establishing a ‘tumor-macrophage’ positive feedback loop (54). TAMs can also suppress T cell-mediated anti-tumor immune responses by expressing immune checkpoint molecules such as PD-L1, thereby facilitating tumor immune evasion (64). Therefore, targeting the recruitment or function of TAMs may represent a novel strategy for bladder cancer immunotherapy.

T cell exhaustion is a pathological state characterized by the progressive loss of effector function in T cells within the TME. It features elevated expression of immune checkpoint molecules, reduced cytokine secretion, and diminished proliferative capacity, serving as a key driver of immune escape in bladder cancer, particularly in MIBC. The bladder cancer TME induces and sustains T cell exhaustion through multiple signaling networks: TAMs highly express PD-L1, which engages PD-1 on CD8^+^ T cells, suppresses the PI3K/AKT signaling pathway, and inhibits T cell activation (65). IL-10 and TGF-β secreted by TAMs directly induce PD-1 expression on CD8^+^ T cells and impair their cytotoxicity. Myeloid-derived suppressor cells (MDSCs) produce reactive oxygen species and arginase, disrupting T cell metabolic homeostasis and accelerating exhaustion (66). The hypoxic TME in bladder cancer activates HIF-1α, upregulating PD-L1 expression on tumor cells while impairing glucose uptake in CD8^+^ T cells. Epigenetically, overexpression of HDACs suppresses the transcription of T cell effector genes and stabilizes the exhausted phenotype (48). T cell exhaustion directly affects patient prognosis and treatment response; MIBC patients with elevated proportions of terminally exhausted CD8^+^ T cells in tumor tissues exhibit significantly shorter OS (67).

TAMs are central orchestrators of the immunosuppressive tumor microenvironment in bladder cancer. Their dual roles in promoting angiogenesis, extracellular matrix remodeling, and T-cell exhaustion make them a promising therapeutic target. Future strategies aimed at reprogramming or depleting TAMs, especially in combination with immune checkpoint inhibitors, hold significant potential to reverse immune evasion and improve treatment outcomes.

Cancer-associated fibroblasts (CAFs) are the most abundant stromal cells in the bladder cancer TME, and their subtype diversity underlies heterogeneous roles in tumor progression. Bladder cancer CAFs can be classified into three core subtypes, each contributing to immune evasion and tumor progression through distinct mechanisms. Inflammatory CAFs (iCAFs) recruit MDSCs via secretion of CXCL1 and IL-6; the latter further activate CAFs through IL-1β secretion, collectively fostering an immunosuppressive microenvironment (68). In NMIBC patients unresponsive to BCG therapy, the proportion of iCAFs is significantly elevated, potentially leading to treatment failure by inhibiting CD8^+^ T cell infiltration (69). Myofibroblastic CAFs (myCAFs) are primary mediators of ECM remodeling. They secrete collagen I and fibronectin to increase stromal stiffness and activate the integrin/α2β1/PI3K/AKT pathway, promoting bladder cancer cell proliferation. Additionally, TGF-β1 secreted by myCAFs directly induces EMT in tumor cells, enhancing invasiveness (70). Studies indicate that high abundance of this subtype is associated with increased postoperative recurrence in MIBC patients (71). Perivascular CAFs interact with endothelial cells via PDGFR-β expression, upregulate angiogenic factors such as VEGF and ANG, and promote tumor microvascular formation. They also enhance vascular barrier function through secreted biomarkers, limiting the penetration of chemotherapeutic agents and immune cells into the tumor core and contributing to therapy resistance (72).

Angiogenesis and stromal remodeling are critical processes in the formation of the TME. Overexpression of pro-angiogenic factors such as VEGF and FGF promotes the formation of new blood vessels within bladder tumor tissue. This neovascularization provides nutrients to tumor cells, while the aberrant vascular architecture simultaneously offers an avenue for tumor cell dissemination (73).

Dysregulated expression of immune checkpoint molecules, such as PD-1/PD-L1, represents a major mechanism of immune evasion in bladder cancer. Clinical studies demonstrate that PD-L1 expression in bladder cancer tissues correlates significantly with tumor stage, grade, and prognosis. Furthermore, PD-L1-positive bladder cancer patients exhibit significantly higher response rates to PD-1/PD-L1 inhibitor therapy compared to PD-L1-negative patients (74). Mechanistically, bladder cancer upregulates PD-L1 expression through multiple pathways, including IFN-γ signaling induction, genetic mutations, and epigenetic modifications. PD-L1 binds to PD-1 on the surface of T cells, suppressing T cell activation and cytotoxicity, thereby enabling evasion of immune surveillance (64, 74). Bladder cancer cells also secrete inhibitory cytokines such as IL-10 and TGF-β, fostering an immunosuppressive microenvironment that synergizes with PD-L1-mediated immune evasion (74). Additionally, dysregulated expression of other immune checkpoint molecules, including CTLA-4, TIM-3, and LAG-3, contributes to the immune escape process in bladder cancer (75). The clinical application of immune checkpoint inhibitors has transformed the therapeutic landscape for bladder cancer. For instance, atezolizumab, the first PD-L1 inhibitor approved, demonstrates significant efficacy in platinum-resistant advanced bladder cancer, achieving an ORR of 23% (76). These advances underscore the critical importance of the tumor microenvironment and immune evasion mechanisms in the molecular pathology of bladder cancer, providing a theoretical foundation for the precision application of immunotherapy.

### 2.6 Metabolic reprogramming

Metabolic reprogramming, whereby tumor cells alter their metabolic pathways to meet the demands of rapid proliferation, represents a hallmark feature distinguishing them from normal cells. Bladder cancer cells exhibit profound metabolic abnormalities, including the reprogramming of glucose, lipid, and amino acid metabolism.

Glucose metabolic reprogramming in bladder cancer manifests as enhanced aerobic glycolysis (the Warburg effect), wherein tumor cells preferentially utilize glycolysis for energy production even under aerobic conditions. This process is primarily regulated by key enzymes such as hexokinase 2 (HK2), phosphofructokinase 1 (PFK1), and pyruvate kinase M2 (PKM2) (77). PKM2, which is highly expressed in bladder cancer, promotes tumor cell proliferation by modulating both glycolysis and gene transcription (78). Lipid metabolic reprogramming in bladder cancer is characterized by increased fatty acid synthesis and inhibition of oxidative phosphorylation. Fatty acid synthase (FASN) is overexpressed in bladder cancer, catalyzing the synthesis of long-chain fatty acids from acetyl-CoA to provide lipid building blocks for tumor cell proliferation. Concurrently, the downregulation of genes involved in fatty acid oxidation leads to diminished fatty acid oxidation capacity in tumor cells (79). Aberrations in amino acid metabolism also contribute to the development and progression of bladder cancer. For example, enhanced glutaminolysis in bladder cancer cells provides carbon and nitrogen sources to support tumor cell proliferation. Additionally, alterations in branched-chain amino acid metabolism within tumor cells are associated with cancer progression (80).

The core signaling pathway interplay network in bladder cancer is presented in **Table 1**.

**Table 1:** Core signaling pathway interplay network in bladder cancer.

Pathway	Mutation frequency	Regulatory mechanism	Downstream effects	References
FGFR3	NMIBC: 60% MIBC: 15%	Mutations/amplification → Activates PI3K/AKT and RAS-MAPK pathways	Promotes proliferation, inhibits apoptosis, induces EMT	(9–12)
TP53/RB1	TP53: MIBC 50-60% RB1: Prevalent in basal subtype	TP53 mutation → Cell cycle dysregulation, DNA repair defects; RB1 loss → E2F3 amplification drives cell cycle progression	Genomic instability, chemoresistance	(15, 16)
PI3K/AKT/mTOR	50%	PIK3CA mutation or PTEN loss	Inhibits apoptosis, promotes angiogenesis	(11, 17, 18)
RAS-MAPK	KRAS: 5-15% NRAS: 2-5%	NRAS/KRAS mutation, cooperates with PI3K/AKT pathway	Promotes proliferation, aberrant differentiation	(10, 11, 20)
Hippo	60-70%	Mutations in GNA13; overexpression of NUAK2; integrin–FAK–CDC42–PP1A axis	Malignant transformation; transition from NMIBC to MIBC; chemoresistance	(21–25)
PPARG	Luminal: 20-25% BASQ: low expression	In luminal: PPARG-RXR heterodimer activation; In BASQ: MEK/ERK phosphorylation and degradation	Luminal: differentiation, cell cycle arrest; BASQ: rapid progression	(28–30)
SHH	Higher frequency in MIBC vs. NMIBC	Gli3 accumulation; crosstalk with PI3K/AKT pathway	Cell proliferation, migration, EMT	(31–34)
Immune checkpoints	–	PD-L1 upregulation (IFN-γ induction/epigenetic); TAM-secreted CCL2/VEGF-C	Suppresses T-cell activity, promotes lymphatic metastasis	(54, 74)
Metabolic reprogramming	Ubiquitous	Glycolysis, lipogenesis	Warburg effect, enhanced proliferation	(78, 79)

## 3 Advances in translational medicine: from molecular mechanisms to precision therapy

### 3.1 Molecular subtyping and clinical significance

Molecular typing serves as the foundation for precision therapy in bladder cancer. Based on large-scale multi-omics studies (such as The Cancer Genome Atlas project), an internationally consensus molecular classification system has been established for MIBC, comprising Luminal-papillary, Luminal-infiltrated, Luminal, Basal/Squamous, and Neuronal subtypes (81). The Luminal subtype is characterized by high expression of urothelial differentiation markers (e.g., Uroplakins, FOXA1) (82). The Luminal-papillary subtype frequently harbors FGFR3 mutations and CDKN2A deletions, is associated with a favorable prognosis, and demonstrates sensitivity to FGFR inhibitors. The Luminal-infiltrated subtype is enriched with immune cell infiltration and may benefit from immunotherapy (83). The Basal/Squamous subtype expresses basal cell markers and squamous differentiation markers, exhibits a high TP53 mutation rate and strong invasiveness; this subtype is sensitive to platinum-based chemotherapy but prone to immune evasion and has a poor prognosis (84). The rare Neuronal subtype expresses neuroendocrine markers (e.g., SYN) and commonly presents with concurrent TP53 and RB1 mutations; it shows heterogeneous responses to immunotherapy, necessitating the exploration of novel targeted strategies, and is associated with an extremely poor prognosis (82). NMIBC also exhibits multiple molecular classification systems. For instance, the UROMOL 2021 classification categorizes NMIBC into Class 1, 2a, 2b, and 3 (82). Class 1 often carries FGFR3 mutations, responds well to BCG therapy, and has the most favorable prognosis. Class 2 most closely resembles MIBC at the molecular level, with Class 2a exhibiting an APOBEC mutational signature and Class 2b expressing PD-L1, indicating potential sensitivity to immunotherapy. Class 3 expresses basal-like markers and stem cell-related genes and is associated with a poorer prognosis (82, 84). **Table 2** presents the molecular subtyping of bladder cancer and clinical significance.

**Table 2:** Molecular subtyping of bladder cancer and clinical significance.

Subtype	Molecular subtype	Key molecular features	Treatment sensitivity	Prognosis	References
MIBC	Luminal**-**papillary	FGFR3 mutations (80%), CDKN2A deletion	Sensitive to FGFR inhibitors (Erdafitinib)	Favorable	(81–84)
	Luminal**-**infiltrated	Abundant tumor-infiltrating immune cells	Responsive to PD-1/PD-L1 inhibitors	Intermediate	
	Luminal	High expression of urothelial differentiation markers (UPK1A/UPK2)	Response to chemotherapy and immunotherapy requires evaluation of other molecular features	Intermediate	
	Basal/Squamous	TP53 mutations (>80%), high expression of basal cell markers	Sensitive to platinum-based chemotherapy	Poor (high metastatic potential)	
	Neuronal	Concurrent TP53/RB1 mutations, SYN expression	Chemoresistant, no standard targeted regimen	Very poor	
NMIBC	Class 1, 2a, 2b, 3	Class 1: FGFR3 mutation; 2a: APOBEC signature; 2b: High PD-L1 expression; 3: UPK expression	Class 1: BCG-sensitive; 2a: Chemotherapy priority; 2b: Immunotherapy-sensitive	Class 1 > 3 > 2b > 2a	

Tumor heterogeneity (ITH) refers to the genetic, phenotypic, and microenvironmental diversity within a single tumor and is a key factor contributing to inaccuracies in molecular subtyping, therapy resistance, and prognostic miscalibration. Bladder cancer commonly exhibits significant ITH, which impacts molecular classification primarily through spatial heterogeneity (subclonal variations across different tumor regions), temporal heterogeneity (therapy-induced subtype switching), and microenvironmental heterogeneity (variations in immune/stromal cell infiltration) (81, 84). For example, bladder cancers often display multifocal growth and regional differentiation variations; a single tumor may contain coexisting ‘Luminal-papillary’ and ‘Basal/Squamous’ subclones. Platinum-based chemotherapy can induce APOBEC-mediated mutation bursts, leading to transdifferentiation across subtypes (84). The presence of such heterogeneity may render single biopsies inadequate for comprehensively capturing the global molecular profile of the tumor, thereby compromising the accuracy and clinical reproducibility of molecular subtyping. Therefore, clinical practice should incorporate strategies such as multi-region sampling or liquid biopsy to improve subclassification reliability and develop combination therapies targeting heterogeneous cell populations.

### 3.2 Breakthroughs in targeted therapy and clinical applications

With in-depth research into the molecular mechanisms of bladder cancer, targeted therapy has emerged as a critical component of precision therapy for this malignancy. In recent years, targeted agents directed against key pathways such as FGFR, PI3K/AKT/mTOR, and VEGF have demonstrated significant progress in clinical investigations. Notably, several of these agents have received approval from regulatory authorities, including the FDA or China’s National Medical Products Administration (NMPA), for the treatment of bladder cancer.

Targeted therapy demonstrates promising clinical prospects in bladder cancer. Given the pivotal role of the FGFR pathway in the pathogenesis and progression of bladder cancer, the development of FGFR inhibitors has become a major focus in its targeted treatment. FGFR3 alteration testing has now been incorporated into the most recent European Association of Urology guidelines, primarily to guide precision treatment and prognostic evaluation of UC (3). Erdafitinib is the first FGFR inhibitor approved by the FDA for the treatment of locally advanced or metastatic UC harboring FGFR2/3 alterations (14). This orally administered multi-targeted tyrosine kinase inhibitor exerts its anti-tumor effects by inhibiting the tyrosine kinase activity of FGFR1-4, thereby blocking FGFR signaling and suppressing tumor cell proliferation. Clinical trials have demonstrated that in platinum-resistant bladder cancer patients harboring FGFR mutations, erdafitinib achieved an ORR of 40%, which was significantly higher than the 11.5% observed with standard chemotherapy, along with a median progression-free survival (PFS) of 5.5 months (13, 14). Furthermore, comparative efficacy analyses of erdafitinib versus chemotherapy (vinflunine or docetaxel) or pembrolizumab in FGFR-altered bladder cancer revealed a significant OS advantage for erdafitinib (median OS: 11.3 months vs. 7.3 months), further substantiating its clinical utility (14, 85). Erdafitinib has consequently been incorporated into the National Comprehensive Cancer Network (NCCN) guidelines as a second-line therapeutic option for advanced FGFR-mutant bladder cancer (86). Beyond erdafitinib, other FGFR inhibitors show considerable promise. Pemigatinib demonstrated an ORR of 23.9% in FGFR3-mutated bladder cancer patients within the phase II FIGHT-201 trial (87). Rogaratinib and derazantinib have also shown encouraging clinical activity in bladder cancer studies (86). Collectively, these advancements establish the clinical application of FGFR inhibitors as a paradigm of translational success in bladder cancer, effectively bridging fundamental molecular mechanisms with precision therapy.

Research on inhibitors targeting the PI3K/AKT/mTOR pathway and VEGF inhibitors has also achieved notable progress in bladder cancer. Temsirolimus, an mTOR inhibitor approved by the FDA for advanced renal cell carcinoma, has demonstrated modest antitumor activity in clinical studies for bladder cancer (88). Clinical trials indicate that temsirolimus monotherapy achieves an ORR of approximately 10% in advanced bladder cancer, with combination chemotherapy potentially enhancing efficacy (89). Furthermore, PI3K inhibitors such as buparlisib and AKT inhibitors such as ipatasertib are currently under investigation in clinical trials for bladder cancer (90). VEGF-targeted therapy in bladder cancer primarily focuses on anti-angiogenesis. Bevacizumab, a recombinant humanized monoclonal antibody against VEGF, is approved for treating various solid tumors. In bladder cancer, combining bevacizumab with chemotherapy (e.g., gemcitabine plus cisplatin) extends OS (91). Clinical studies report that bevacizumab plus chemotherapy achieves a median OS of 12.3 months in metastatic bladder cancer, superior to chemotherapy alone (92). Additionally, other VEGF inhibitors, including apatinib and ramucirumab, have shown encouraging clinical activity in bladder cancer studies (73).

Overexpression or amplification of human epidermal growth factor receptor 2 (HER2) in bladder cancer correlates with tumor progression and poor prognosis, particularly being more prevalent in lymph node metastases (93). Consequently, HER2-targeted therapy has emerged as another promising area of translational medicine for bladder cancer. Disitamab vedotin, a HER2-targeted antibody-drug conjugates (ADCs), links an anti-HER2 antibody to the cytotoxic payload monomethyl auristatin E (MMAE). This agent has demonstrated significant efficacy in clinical trials. Results from a phase II clinical trial involving patients with HER2-positive locally advanced or metastatic bladder cancer showed that disitamab vedotin achieved an ORR of 50.5%, including a complete response (CR) rate of 9.8%, and a median PFS of 6.9 months (94). Disitamab vedotin has now received approval in China for the treatment of HER2-positive advanced bladder cancer, becoming the first ADC therapy specifically targeting HER2 in this malignancy (94). Another phase III clinical trial evaluating trastuzumab emtansine (T-DM1) in HER2-positive bladder cancer was terminated early due to enrollment challenges. Nevertheless, it demonstrated an ORR of 38.5%, suggesting the potential of HER2-targeted approaches (94). Beyond HER2-targeted ADCs, enfortumab vedotin—a Nectin-4-targeted ADC conjugated with MMAE—has revolutionized the treatment of advanced UC. The phase III EV-301 trial showed that enfortumab vedotin monotherapy achieved a median OS of 12.8 months and an ORR of 40% in patients refractory to platinum and PD-(L)1 inhibitors (95, 96). In first-line settings, the combination of enfortumab vedotin with pembrolizumab yielded an ORR of 73% in advanced MIBC, leading to FDA approval as a first-line option (96). Sacituzumab govitecan, a Trop-2-targeted ADC loaded with SN-38, demonstrated an ORR of 27% and median OS of 10.9 months in the TROPHY-U-01 trial for platinum/PD-(L)1-refractory UC. In BCG-unresponsive NMIBC, it achieved a 35% 6-month CR rate, supporting its potential in non-muscle-invasive disease (95). Collectively, these studies indicate that HER2, Nectin-4, and Trop-2, as key driver targets in bladder cancer, have seen their targeted therapeutics successfully transition from laboratory research to clinical application. This progression offers new treatment options for patients with corresponding biomarker-positive disease.

### 3.3 Innovations in immunotherapy and clinical practice

The advent of immunotherapy has revolutionized the treatment landscape for bladder cancer. Particularly, the application of immune checkpoint inhibitors (ICIs) represents a major breakthrough in translational medicine for this malignancy and has become a cornerstone therapeutic option for advanced disease.

Currently, PD-1/PD-L1 inhibitors demonstrate significant efficacy in both first-line and second-line settings for bladder cancer, fundamentally altering its management paradigm. Pembrolizumab was the first PD-1 inhibitor approved by the FDA for the treatment of platinum-resistant locally advanced UC (97). The KEYNOTE-045 clinical trial demonstrated that pembrolizumab monotherapy achieved an ORR of 21.1% and a median OS of 10.3 months in advanced bladder cancer, superior to conventional chemotherapy (90). Furthermore, pembrolizumab is also approved for the treatment of BCG-unresponsive NMIBC (98). Nivolumab, another pivotal PD-1 inhibitor, obtained FDA approval for platinum-refractory advanced UC based on the phase II CheckMate 275 trial. The study revealed an ORR of 19.6%, a median OS of 8.7 months, and durable responses in 16% of patients at 12 months (99). In BCG-unresponsive NMIBC, the combination of nivolumab and linrodostat achieved an ORR of 32%, highlighting its potential in salvage therapy (100). Tislelizumab, a humanized PD-1 antibody, has shown promising outcomes in MIBC combined with chemoradiotherapy. A real-world study involving 25 MIBC patients reported a 92% clinical CR rate and 96% 1-year OS, supporting its role in bladder-preserving strategies (95). Camrelizumab, administered intravesically for BCG-failure high-risk NMIBC, achieved a 12-month recurrence-free survival rate of 42% with favorable safety profiles (95).

Atezolizumab was the first PD-L1 inhibitor approved by the FDA for locally advanced or metastatic UC progressing after platinum-based chemotherapy (76). The phase II IMvigor210 clinical trial reported an ORR of 14.8% for atezolizumab in second-line bladder cancer, with the ORR reaching 26.0% in PD-L1-positive patients. Subsequently, the phase III IMvigor211 trial further confirmed that atezolizumab improved OS compared to chemotherapy in the second-line setting, with a more pronounced benefit observed in PD-L1-positive patients (101). Additionally, other PD-L1 inhibitors, including durvalumab and avelumab, have also gained regulatory approvals for bladder cancer treatment based on clinical trial evidence, expanding the available therapeutic options (102, 103). Sasanlimab exhibited enhanced efficacy when combined with BCG in BCG-naive high-risk NMIBC, with a 3-year recurrence-free survival rate of 71% (95). Durvalumab combined with BCG in BCG-relapsed NMIBC achieved an 85% 6-month CR rate (96), while avelumab as first-line maintenance therapy for advanced UC prolonged median OS to 23 months (99). BCG, the standard adjuvant therapy for high-risk NMIBC, exerts its anti-tumor effects through mechanisms closely linked to immune modulation. BCG activates both innate and adaptive immune responses, and its efficacy correlates strongly with immune cell infiltration within the TME and the release of specific cytokines (104). For patients with BCG-unresponsive NMIBC, PD-1/PD-L1 inhibitors provide a crucial novel treatment alternative (105). Notably, analyses of the tumor microenvironment in BCG-unresponsive tumors reveal a critical yet paradoxical phenomenon: these tumors often exhibit significant immune cell infiltration (e.g., T cells and macrophages), resulting in an ‘immune-rich but dysfunctional’ state. This infiltration is likely triggered by BCG-induced immune stimulation; however, tumors achieve immune escape through mechanisms such as upregulating immune checkpoints (e.g., PD-L1, LAG-3, TIM-3), recruiting immunosuppressive cells (e.g., M2 macrophages, Tregs), and inducing T-cell exhaustion (104). Thus, BCG-unresponsive tumors are not ‘immune deserts’ but rather exist in a ‘pre-activated yet suppressed’ state, rendering them highly sensitive to ICIs. ICIs can block inhibitory signals and reactivate infiltrated but functionally impaired T cells, thereby exerting antitumor effects. This explains why PD-1/PD-L1 inhibitors demonstrate significant efficacy in patients with BCG-unresponsive NMIBC (105). Therefore, the immune infiltration status following BCG treatment may serve as a positive biomarker for predicting ICI response.

Despite the remarkable reshaping of the bladder cancer treatment landscape by ICIs, their clinical application continues to encounter challenges and opportunities. Future research should focus on elucidating the mechanisms underlying primary and acquired resistance, such as the heterogeneity of the TME, compensatory upregulation of alternative immunosuppressive pathways, and defects in antigen presentation machinery. Concurrently, identifying predictive biomarkers beyond PD-L1 (e.g., TMB, immune gene signatures, the microbiome) is crucial for precisely selecting the patient populations most likely to benefit. Furthermore, novel combination strategies—integrating ICIs with other immunomodulators, targeted therapies, antibody-drug conjugates, or radiotherapy—hold promise for synergistically overcoming immunosuppression and expanding the beneficiary population. Ultimately, translating these scientific insights into effective clinical regimens will offer new hope for patients with bladder cancer.

### 3.4 Exploration and optimization of combination therapy strategies

Given the complexity and heterogeneity of the molecular mechanisms underlying bladder cancer, single therapeutic modalities often fail to achieve optimal outcomes. Consequently, combination therapy strategies have emerged as a critical direction for advancing bladder cancer treatment. Currently, various combination approaches, including targeted therapy with immunotherapy, chemotherapy with immunotherapy, and combinations of different targeted agents, are actively being explored in clinical research.

The combination of targeted therapy and immunotherapy represents a major current research focus in bladder cancer treatment. For instance, preclinical studies demonstrate synergistic antitumor effects from combining FGFR inhibitors with PD-1/PD-L1 inhibitors (86). Clinical trials indicate that the combination of erdafitinib and pembrolizumab achieves an ORR exceeding 50% in patients with FGFR-altered bladder cancer, highlighting its promising clinical potential (85). Combining chemotherapy with immunotherapy has shown significant advantages in the first-line treatment of bladder cancer. The KEYNOTE-361 clinical trial reported a median OS of 17.0 months for pembrolizumab combined with gemcitabine and cisplatin in metastatic bladder cancer, superior to chemotherapy alone (106). Furthermore, atezolizumab combined with chemotherapy has also demonstrated a trend towards prolonged survival in clinical trials (107). Additionally, integrating local and systemic therapies is crucial for the comprehensive management of bladder cancer. For MIBC patients, neoadjuvant immunotherapy combined with radical cystectomy has emerged as an important treatment paradigm. Studies show that neoadjuvant PD-1/PD-L1 inhibitors can induce pathological CR in a subset of MIBC patients, improving their prognosis (108). For NMIBC patients, combining BCG with PD-1 inhibitors may enhance treatment response rates (98).

Although combination therapies represent a promising advance in bladder cancer treatment, their optimization and clinical application still face significant challenges. There is a need to strengthen biomarker discovery through multi-omics technologies to identify molecular features (e.g., TMB, specific genetic alterations, and immune microenvironment phenotypes) that predict response to combination regimens, enabling precise patient stratification. Exploring novel combination strategies beyond current paradigms—such as integrating immunotherapy with ADCs, metabolic pathway inhibitors, or localized radiotherapy—may help overcome tumor heterogeneity and therapy resistance. Furthermore, elucidating the mechanisms of resistance to combination therapies (e.g., emerging immune escape pathways or adaptive responses to targeted agents) and expanding research to encompass more bladder cancer subtypes (e.g., distinct molecular classes or rare variants) is crucial. Ultimately, integrating fundamental research insights with real-world evidence will be essential to translate these advances into tangible benefits for patients, representing a central goal in the field of combination therapy for bladder cancer.

### 3.5 Gene therapy and precision intervention

Gene therapy, as a frontier field in translational medicine, demonstrates unique advantages in bladder cancer treatment. Nadofaragene firadenovec is an adenoviral vector-based gene therapeutic. Its mechanism involves the adenovirus delivering the human interferon α-2b gene to bladder cancer cells, inducing antiviral and antitumor immune responses while modulating the tumor microenvironment. Approved by the FDA for the treatment of BCG-unresponsive NMIBC, phase III clinical trials demonstrated a CR rate of 53.4%, with response durations lasting up to 12 months. Common adverse events included local reactions such as urinary frequency, urgency, and hematuria, while systemic toxicity was low, indicating a favorable safety profile (109). This advancement signifies a breakthrough in the clinical translation of gene therapy for bladder cancer, offering a novel therapeutic option for BCG-unresponsive patients.

CRISPR-Cas9 gene editing technology also exhibits immense potential in both fundamental research and translational applications for bladder cancer. *In vitro* and animal models have confirmed that targeted knockout of oncogenes such as FGFR3 or Gαi3 using CRISPR-Cas9 inhibits tumor growth (19, 110). Knocking out Gαi3 also significantly suppresses bladder cancer cell proliferation and invasion while enhancing chemosensitivity (19). Furthermore, CRISPR-Cas9 technology can be employed to engineer immune cells, such as chimeric antigen receptor (CAR) T cells, to enhance their ability to recognize and eliminate tumor cells (110). Although the clinical application of CRISPR-Cas9 in bladder cancer remains in its early stages, its advantages in precision targeting and efficient editing foreshadow a significant future role in bladder cancer translational medicine. However, there is a need to develop novel vector systems that are more efficient, highly specific, and less immunogenic (such as synthetic viral vectors or non-viral nanocarriers) to address issues such as low *in vivo* delivery efficiency and off-target effects, particularly improving the *in vivo* stability and tumor specificity of the CRISPR system. And it is essential to actively explore combination strategies that integrate gene therapy with other treatment modalities (e.g., immune checkpoint inhibitors, chemotherapy, or oncolytic viruses) to synergistically enhance anti-tumor immune responses and overcome therapy resistance. Furthermore, given the heterogeneity of bladder cancer, developing personalized gene editing strategies based on molecular subtypes (e.g., simultaneously targeting multiple driver genes or modulating the immune microenvironment) holds significant promise. Only by overcoming these bottlenecks can gene therapy become an integral component of the precision medicine landscape for bladder cancer.

The clinical advances in precision therapeutics for bladder cancer is presented in **Table 3**.

**Table 3:** Clinical advances in precision therapeutics for bladder cancer.

Therapeutic class	Representative agent	Mechanism of action	Approved indication	ORR	References
FGFR inhibitor	Erdafitinib	FGFR1–4 tyrosine kinase	Locally advanced or metastatic UC with FGFR2/3 alterations	40%	(13, 14)
mTOR inhibitor	Temsirolimus	mTOR	locally advanced bladder cancer	10%	(89)
ADCs	Disitamab vedotin	HER2/MMAE	HER2-positive advanced UC; BCG-unresponsive NMIBC	50.5%	(94)
	Trastuzumab emtansine	HER2/DM1	HER2-positive recurrent/metastatic UC	38.5%	
	Enfortumab vedotin	Nectin-4/MMAE	Advanced UC after platinum + ICIs; BCG-unresponsive NMIBC	40%	(95, 96)
	Sacituzumab govitecan	Trop-2/SN-38	Advanced UC after platinum + ICIs	27%	(95)
PD-1 ICIs	Pembrolizumab	PD-1	BCG-unresponsive NMIBC; Platinum-resistant advanced UC	21.1%	(90, 98)
	Nivolumab		Advanced UC; BCG-unresponsive NMIBC	19.6%	(99, 100)
	Tislelizumab		Advanced MIBC combined with chemoradiotherapy	92% (CR)	(95)
	Camrelizumab		High-risk NMIBC (post-BCG failure)	–	(95)
PD-L1 ICIs	Atezolizumab	PD-L1	Platinum-ineligible advanced UC; BCG-unresponsive NMIBC	26%	(101)
	Avelumab		Advanced UC; BCG-unresponsive NMIBC	28%	(99, 103)
	Durvalumab		BCG-unresponsive/relapsed NMIBC; advanced UC	50%	(96)
	Sasanlimab		BCG-naive high-risk NMIBC	–	(95)
Gene therapy	Nadofaragene firadenovec	Adenoviral delivery	BCG-unresponsive NMIBC	53.4% (CR)	(109)

### 3.6 Liquid biopsy and clinical diagnosis

**Figure 1:**
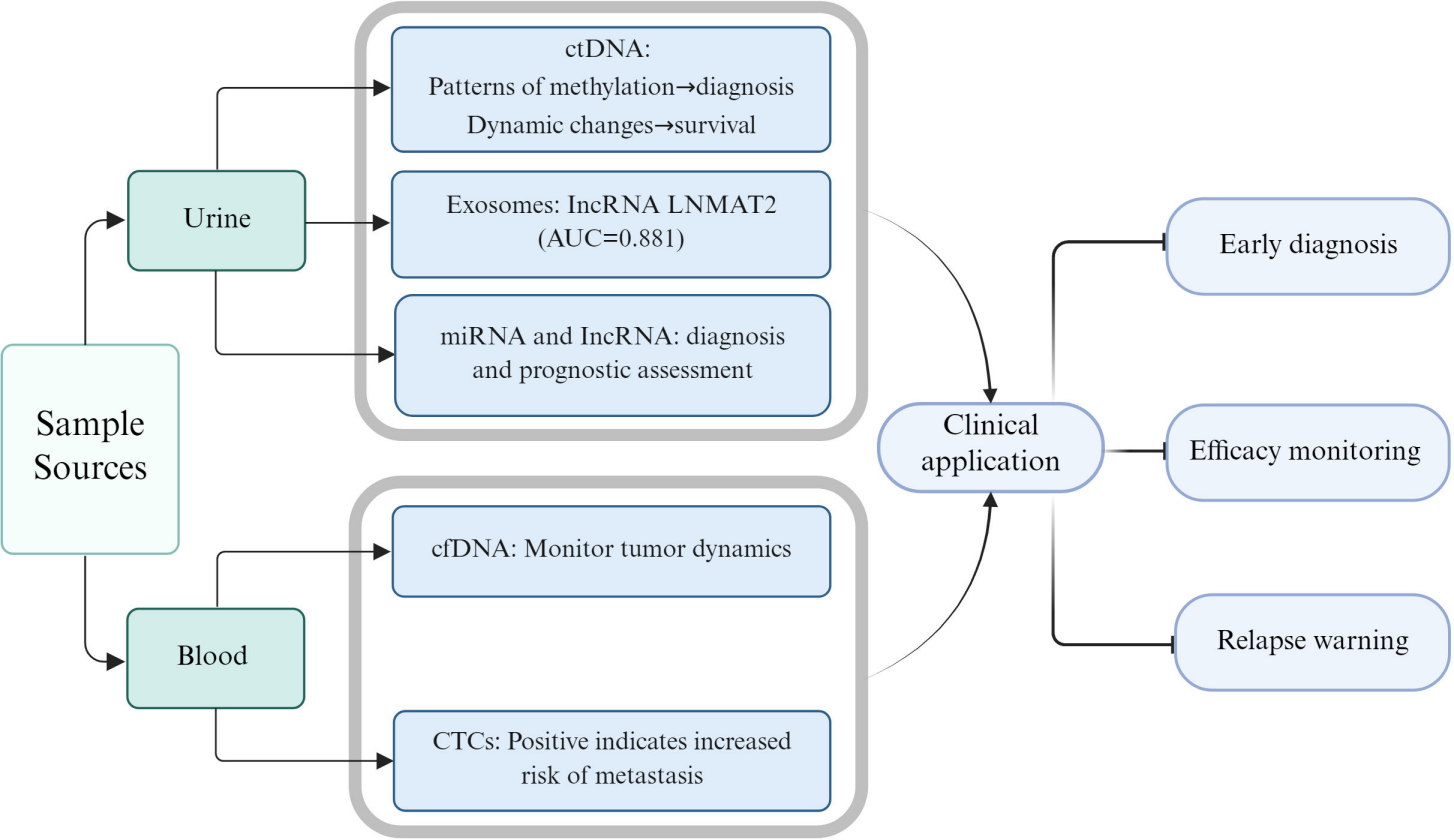
Clinical utility of liquid biopsy in bladder cancer. Urine-based biomarkers include ctDNA (whose methylation patterns aid in early diagnosis and dynamic monitoring), exosomal lncRNA LNMAT2 (a diagnostic marker for BCG-unresponsive NMIBC), miRNAs and lncRNAs. Blood-based biomarkers comprise cfDNA for monitor tumor dynamics and CTCs for assessing metastatic risk. Collectively, liquid biopsy enables non-invasive early diagnosis, real-time efficacy evaluation, and recurrence warning. ctDNA, circulating tumor DNA; cfDNA, cell-free DNA; CTCs, circulating tumor cells; lncRNA, long non-coding RNA.

Liquid biopsy, as a non-invasive molecular diagnostic technique, holds significant clinical value in the precision medicine of bladder cancer, encompassing early diagnosis, treatment efficacy monitoring, and recurrence prediction. The detection of biomarkers, such as circulating tumor cells (CTCs), circulating tumor DNA (ctDNA), and exosomes, in blood, urine, or other bodily fluids enables precise diagnosis and dynamic monitoring of bladder cancer (**Figure 1**).

The detection of biomarkers in urine offers unique advantages for the diagnosis of bladder cancer due to its non-invasive nature and convenience. CtDNA consists of DNA fragments released by tumor cells into the bloodstream, carrying genetic mutation information consistent with the primary tumor. The methylation patterns of ctDNA can be used for early diagnosis; for instance, the methylation levels of genes like FGFR3 and TP53 are closely associated with the presence of bladder cancer, demonstrating high sensitivity and specificity (111). The detection of ctDNA in urine has also been confirmed as a viable method for monitoring bladder cancer recurrence, with its mutational profile showing high concordance with tumor tissue (112). Studies indicate that dynamic changes in ctDNA, such as clearance status, can promptly reflect treatment response in bladder cancer, guiding therapeutic adjustments (113, 114). Conversely, these changes are also associated with recurrence risk: patients without ctDNA clearance after treatment have a significantly higher risk of recurrence compared to those with ctDNA clearance. Furthermore, patients achieving ctDNA clearance experience prolonged survival and improved prognosis (113, 115).

Exosomes, as another critical component of liquid biopsy, carry abundant molecular information in bladder cancer. For example, the lncRNA LNMAT2 in urinary exosomes can serve as a diagnostic biomarker for BCG-unresponsive NMIBC, achieving an area under the curve (AUC) of 0.881 (55). Protein and miRNA profiles within exosomes can also be utilized for bladder cancer grading and staging (116). Additionally, the detection of biomarkers such as miRNA and lncRNA in urine shows promising potential for the diagnosis and prognostic assessment of bladder cancer (117, 118).

In the exploration of clinical applications for urinary biomarkers, the DaBlaCa-15 trial provided high-level evidence of great importance (119). This multicenter randomized noninferiority trial focused on patients with high−risk NMIBC and aimed to evaluate the value of alternating cystoscopy with the Xpert Bladder Cancer Monitor (XBCM)—a urinary biomarker test—during follow−up. Patients were randomly assigned to either an intervention group (XBCM testing every 4 months) or a control group (routine cystoscopy every 4 months). The primary endpoint was recurrence-free survival for high-grade NMIBC, MIBC, or metastatic UC. Results demonstrated noninferiority in recurrence detection in the intervention group compared to the control group at 24 months (risk difference 0.08%, 95% confidence interval -7.3% to 7.4%), while the number of cystoscopies performed was reduced by 55% (119). The XBCM test showed a sensitivity of 91% and a negative predictive value of 99% for high-grade disease. Moreover, in 13 patients with recurrence, the test indicated recurrence a median of 8.3 months earlier than histological confirmation (119). These findings strongly support that urinary biomarker testing can effectively ensure the safety of bladder cancer surveillance while reducing the need for invasive procedures, further underscoring the clinical utility of urinary biomarkers in the management of bladder cancer.

CTCs and cell-free DNA (cfDNA) in blood also possess diagnostic and prognostic value. Studies have shown that the presence of CTCs is associated with metastasis and poor prognosis in bladder cancer, while the mutational profile in plasma cfDNA can be used to monitor tumor dynamics (120). Due to its non-invasiveness and repeatability, liquid biopsy has become an indispensable molecular diagnostic tool for precision management of bladder cancer, particularly applicable for treatment monitoring and recurrence prevention.

## 4 Precision therapy strategies: from multi-omics to clinical decision-making

The rapid advancement of multi-omics technologies has provided unprecedented opportunities for precise prognostic assessment and treatment strategy formulation in bladder cancer. By integrating multi-dimensional data from genomics, transcriptomics, epigenetics, proteomics, metabolomics, and radiomics, researchers can more comprehensively reveal the molecular characteristics of bladder cancer and their association with clinical outcomes, thereby promoting the development of individualized treatment decisions.

In genomics, beyond FGFR3 mutations as important predictive and prognostic markers, other genetic alterations such as TP53, RB1, and PIK3CA also play key roles in the development and progression of bladder cancer (9, 10). For example, TP53 mutations are common in MIBC and are significantly associated with tumor progression and poor prognosis. Additionally, the mutation status of DNA damage repair pathway genes (e.g., ERCC2) can predict sensitivity to platinum-based chemotherapy, providing a basis for adjuvant therapy selection. In recent years, molecular classification based on next-generation sequencing technology (e.g., TCGA classification) has categorized bladder cancer into several subtypes, each with distinct clinical behaviors and treatment responses, laying the foundation for translating basic research into clinical practice (81, 82).

Transcriptomics studies have further enriched our understanding of bladder cancer heterogeneity. For instance, the Lund classification and TCRNA classification systems categorize bladder cancer into different subtypes based on gene expression profiles, which exhibit significant differences in prognosis, immune microenvironment composition, and treatment response. The 20-gene model developed by Smith et al. can preoperatively predict lymph node metastasis status with an AUC of 0.67 (121). Although it has certain predictive ability, its performance in independent validation is limited. In contrast, the KNN51 model developed by Seiler et al., based on whole transcriptome data and including 51 genes (24 of which are non-coding RNAs), achieved an AUC of 0.82 in the validation set, significantly outperforming traditional clinical indicators and previous models (122). This highlights the important role of ncRNAs in predicting lymph node metastasis. These models provide clinicians with powerful molecular tools for preoperative assessment of lymph node metastasis risk, determining the extent of surgery, and deciding whether to administer neoadjuvant chemotherapy.

Epigenetic changes, particularly DNA methylation, play a key role in the development, progression, and prognosis of bladder cancer. Numerous studies have shown that promoter hypermethylation (e.g., of GATA4, RASSF1A, CDH1) is closely associated with tumor suppressor gene silencing, tumor progression, and poor prognosis (19). Conversely, global hypomethylation may lead to genomic instability and oncogene activation. In recent years, epigenetic drugs such as DNA methyltransferase inhibitors and histone deacetylase inhibitors have been applied in hematological malignancies and are undergoing clinical trials in solid tumors (123). For example, demethylating agents such as azacitidine and decitabine have shown inhibitory effects on bladder cancer cells *in vitro* and in animal models and may reverse chemotherapy resistance. Inhibitors of histone modifiers (e.g., EZH2) have demonstrated therapeutic potential in basal-like bladder cancer, especially when combined with immunotherapy (123). These findings not only reveal the important role of epigenetic regulation in bladder cancer but also provide direction for developing new treatment strategies.

Proteomics and metabolomics, by analyzing changes in proteins and metabolites in tumor tissue and body fluids, offer new biomarkers for early diagnosis, prognostic assessment, and treatment response prediction in bladder cancer. For example, elevated levels of Matrix Metalloproteinase-7 in urine are associated with the risk of lymph node metastasis (124). Additionally, numerous studies have reported the value of urinary fibronectin, nuclear matrix protein 22, and peptide biomarkers in the non-invasive diagnosis of bladder cancer. Metabolomics studies have revealed significant metabolic reprogramming in bladder cancer cells, such as enhanced glycolysis, abnormal glutamine metabolism, and altered lipid metabolism (123). These metabolic changes not only facilitate tumor growth and metastasis but also serve as potential therapeutic targets. For instance, targeting key glycolytic enzymes like PKM2 or glutaminase may inhibit tumor progression. Recent studies indicate that urinary metabolite profiles can distinguish NMIBC from MIBC and even predict response to BCG therapy, providing a non-invasive assessment tool for clinical use (123).

Radiomics, by extracting high-throughput quantitative features from conventional images such as CT and MRI and combining them with machine learning methods, has shown great potential in preoperative staging, lymph node metastasis prediction, treatment response assessment, and prognosis evaluation in bladder cancer. Wu et al. developed a CT-based radiomics nomogram that combined imaging features and clinical variables, achieving AUCs of 0.926 and 0.899 in the training and validation sets, respectively, for predicting lymph node metastasis—significantly better than subjective radiological assessment alone (125). Another study based on T2-weighted MRI extracted 718 features, ultimately selecting 9 to construct a signature that achieved AUCs of 0.901 and 0.845 in the training and validation sets, respectively, and maintained good discriminative ability in the clinically node-negative subgroup (AUC = 0.841) (126). These models not only improve the accuracy of preoperative staging but also help identify high-risk patients who may benefit from extended lymph node dissection or neoadjuvant chemotherapy, thereby optimizing treatment decisions. Furthermore, radiomic features have also demonstrated potential value in assessing response to neoadjuvant chemotherapy and predicting recurrence and survival outcomes, offering possibilities for the comprehensive precision management of bladder cancer.

The integration of multi-omics data is an inevitable trend in the future development of precision medicine. By combining genomic, transcriptomic, epigenetic, proteomic, metabolomic, and radiomic data, more comprehensive predictive models can be constructed, improving the ability to resolve disease heterogeneity and ultimately achieving truly individualized treatment. For example, combining molecular subtypes with imaging features can more accurately predict tumor biological behavior; integrating ctDNA detection with dynamic radiomics can be used for real-time monitoring of treatment response and resistance. Although multi-omics integration in bladder cancer is still in its exploratory stages, advances in computational biology and artificial intelligence methods, along with large prospective cohort studies, hold promise for making multi-omics-guided precision therapy strategies a standard in clinical practice in the near future.

## 5 Discussion

Despite significant advances in the molecular mechanism research and translational applications of bladder cancer, precision medicine still faces multiple complex challenges. These challenges are rooted in both the biological characteristics of the tumor itself and systemic barriers in translating basic research into clinical practice.

Firstly, the high heterogeneity of bladder cancer remains a core bottleneck for implementing precision therapy. This heterogeneity manifests across multiple dimensions: spatially, tumors often exhibit multifocal growth, with different molecular subtypes such as ‘luminal papillary’ and ‘basal/squamous’ subclones coexisting within the same lesion—for example, some MIBC patients harbor both FGFR3-mutated luminal subpopulations and TP53/RB1 double-mutated basal subpopulations, making it difficult for a single biopsy to fully capture the tumor’s molecular landscape (81, 84); temporally, therapeutic interventions drive subclonal evolution, such as platinum-based chemotherapy inducing APOBEC-mediated mutation bursts promoting transformation from luminal to more aggressive basal subtypes (84); in the tumor microenvironment dimension, regional variations exist in immune cell infiltration, stromal stiffness, and cytokine networks—for instance, BCG-unresponsive NMIBC shows a significant increase in inflammatory CAFs, fostering a localized immunosuppressive microenvironment (69). This multidimensional heterogeneity directly contributes to diverse molecular targets and individual variations in treatment response (127, 128), rendering a ‘one-size-fits-all’ approach ineffective. Achieving true individualized precision therapy remains a challenge.

Secondly, the emergence and evolution of treatment resistance severely constrain the durability of efficacy, encompassing both primary and acquired resistance. In targeted therapy, mechanisms of FGFR inhibitor resistance are increasingly understood: some patients develop FGFR3 secondary mutations (e.g., V564M) reducing drug-binding affinity, while others activate bypass pathways such as PI3K/AKT/mTOR or RAS-MAPK (86). Clinical data show that FGFR3-mutated patients with concurrent PI3K pathway activation have a more than 30% lower response rate to erdafitinib (14). In immunotherapy, resistance mechanisms are more complex: primary resistance is associated with an ‘immune-desert’ tumor microenvironment (74), as seen in luminal subtype bladder cancers where PPARG pathway activation suppresses NF-κB signaling, resulting in low PD-L1 expression and insufficient T-cell infiltration (30); acquired resistance often involves defects in antigen presentation machinery (e.g., B2M mutation), upregulation of alternative immune checkpoints (e.g., TIM-3/LAG-3), or aberrant activation of the Hippo-YAP pathway, which recruits MDSCs via the IL-6/STAT3 axis to reinforce immunosuppression (21–23). Additionally, chemotherapy resistance is closely linked to abnormalities in DNA damage repair pathways (e.g., loss of ERCC2 mutations) and metabolic reprogramming (e.g., PKM2-mediated enhanced glycolysis) (77), with PTEN-deficient tumors showing a 30% higher cisplatin resistance rate than wild-type tumors (17). In-depth analysis of the dynamic evolution of resistance and the development of targeted reversal strategies are urgent clinical priorities.

Thirdly, the clinical translation efficiency of biomarkers remains low, with many potential markers struggling to enter clinical decision-making systems. Although basic research has identified numerous promising molecular markers, such as urinary lncRNA LNMAT2 (55) and plasma ctDNA methylation sites (e.g., FGFR3, TP53) (111), most remain in small-sample validation stages. Key obstacles include: insufficient specificity and sensitivity (124)—for example, urinary miR-21 alone has an AUC of only 0.72 for early NMIBC diagnosis, necessitating multi-marker panels (117); lack of standardized detection systems, with variations in ctDNA extraction methods and detection thresholds across laboratories leading to poor result comparability (112); and inadequate clinical utility validation, as most markers have not been confirmed through multicenter, prospective cohort studies for their value in guiding treatment decisions—for instance, while TMB’s prognostic value in advanced bladder cancer is established, its predictive efficacy in NMIBC remains unclear (39, 40). Establishing a standardized pipeline from biomarker discovery to clinical validation is a critical task for translational medicine.

Lastly, advancing precision therapy relies on deep interdisciplinary collaboration, yet current synergistic mechanisms among disciplines remain underdeveloped. Precision therapy for bladder cancer requires integration of resources from basic medicine (molecular biology, immunology), clinical medicine (urology, medical oncology), bioinformatics (multi-omics data analysis), and data science (AI modeling) (129, 130). However, existing collaborative models often face barriers: basic research frequently focuses on single molecular mechanisms, disengaged from clinical needs—for example, some SHH pathway inhibitors showed efficacy in animal models but failed clinically due to unaddressed cross-activation with the PI3K pathway (34); clinical studies often lack dynamic tracking of molecular changes pre- and post-treatment, hindering insights into resistance mechanisms; bioinformatics models are mostly built on retrospective data and lack prospective validation—e.g., some transcriptome-based lymph node metastasis prediction models saw AUC drop from 0.82 to 0.65 in external cohorts (122). Insufficient data sharing and misaligned research goals across disciplines somewhat constrain the coordinated development of precision therapy.

Future research in precision therapy for bladder cancer should focus on the following key directions:

Deepening dynamic analysis of treatment resistance mechanisms: Utilize longitudinal multi-omics technologies (serial genomic, transcriptomic, and proteomic analyses) to track the evolution of tumor molecular characteristics during treatment, mapping resistance pathways. Develop targeted strategies for different resistance types: for FGFR inhibitor resistance, explore dual FGFR/PI3K inhibitors or combination with MET inhibitors; for immune checkpoint inhibitor resistance, combine epigenetic modulators (e.g., HDAC inhibitors) to reverse T-cell exhaustion or use ADC drugs to enhance antigen release. For instance, preclinical studies show that combining erdafitinib and pembrolizumab synergistically inhibits FGFR signaling and alleviates immune suppression, increasing the ORR in FGFR-mutated patients to over 50%.

Accelerating clinical translation and standardization of biomarkers: Conduct large-sample, multicenter prospective cohort studies to systematically validate the diagnostic, prognostic, and predictive values of biomarkers. Establish standardized detection platforms, such as uniform reagents, instruments, and thresholds for urinary ctDNA methylation testing, and develop non-invasive diagnostic systems integrating urinary markers and radiomics—e.g., the XBCM testing system validated in the DaBlaCa-15 trial reduced cystoscopy frequency by 55% while maintaining 91% sensitivity for high-grade recurrence detection. Build clinical decision-support systems based on biomarkers, such as predictive models for immunotherapy benefit combining TMB, PD-L1 expression, and molecular subtypes, enabling precise patient stratification.

Promoting in-depth application of artificial intelligence in precision therapy: Develop machine learning models based on multi-omics data to enable automated treatment recommendations from molecular features. For example, use deep learning to analyze correlations between CT/MRI radiomic features and genomic data, constructing models for preoperative prediction of lymph node metastasis with AUCs exceeding 0.92 to inform surgical decisions; train AI models on real-world data to predict response rates and resistance risks for FGFR inhibitors, ADC drugs, etc., aiding clinical drug selection. Simultaneously, enhance research on AI model interpretability to avoid ‘black-box’ decision-making.

Optimizing development and validation of multi-target combination therapies: Use systems biology approaches to predict drug synergies and design individualized combination regimens. For instance, target metabolic reprogramming features by combining glycolysis inhibitors (e.g., HK2 inhibitors) with PD-1 inhibitors to enhance immunotherapy efficacy by ameliorating metabolic dysregulation in the tumor microenvironment; for HER2-positive patients, combining disitamab vedotin with immune checkpoint inhibitors has shown ORR increased to over 60% in clinical studies. Additionally, focus on toxicity management in combination therapies, balancing efficacy and safety through dose optimization and biomarker guidance.

In conclusion, translational medicine, serving as a bridge connecting basic research and clinical application, plays a pivotal role in the precision therapy of bladder cancer. Future translational research should place greater emphasis on being clinically demand-driven, initiating basic research from clinical questions and then rapidly translating the findings into clinical practice. This bidirectional translation model enhances the relevance and utility of the research. Furthermore, translational medicine should promote the integration and standardization of multi-omics data, establishing cross-platform molecular databases for bladder cancer to provide data support for precision therapy. For example, databases integrating genomic, transcriptomic, proteomic, and clinical data can support the training and validation of machine learning models. Translational medicine should also facilitate the clinical translation of novel therapeutic technologies, such as gene editing, cell therapy, and nanomedicine. For instance, the clinical application of CRISPR-Cas9 technology in bladder cancer requires translational research to address key challenges like delivery efficiency and off-target effects. In conclusion, precision therapy for bladder cancer is currently in a phase of rapid advancement. The deepening understanding of molecular mechanisms and the flourishing development of translational medicine provide unprecedented opportunities for the individualized treatment of bladder cancer. Despite facing numerous challenges, with technological progress and deeper research, precision therapy for bladder cancer will undoubtedly witness new breakthroughs, offering patients longer survival and improved quality of life.

## References

[1] Bray F Laversanne M Sung H Ferlay J Siegel RL Soerjomataram I . Global cancer statistics 2022: GLOBOCAN estimates of incidence and mortality worldwide for 36 cancers in 185 countries. CA Cancer J Clin. (2024) 74:229–63. doi: 10.3322/caac.21834, PMID: 38572751

[2] Siegel RL Giaquinto AN Jemal A . Cancer statistics, 2024. CA Cancer J Clin. (2024) 74:12–49. doi: 10.3322/caac.21820, PMID: 38230766

[3] Van Der Heijden AG Bruins HM Carrion A Cathomas R Compérat E Dimitropoulos K . European association of urology guidelines on muscle-invasive and metastatic bladder cancer: summary of the 2025 guidelines. Eur Urol. (2025) 87:582–600. doi: 10.1016/j.eururo.2025.02.019, PMID: 40118736

[4] Curtius K Wright NA Graham TA . An evolutionary perspective on field cancerization. Nat Rev Cancer. (2018) 18:19–32. doi: 10.1038/nrc.2017.102, PMID: 29217838

[5] Höglund M . On the origin of syn- and metachronous urothelial carcinomas. Eur Urol. (2007) 51:1185–93; discussion 1193. doi: 10.1016/j.eururo.2006.11.025, PMID: 17123702

[6] Sidransky D Frost P Von Eschenbach A Oyasu R Preisinger AC Vogelstein B . Clonal origin of bladder cancer. N Engl J Med. (1992) 326:737–40. doi: 10.1056/nejm199203123261104, PMID: 1445507

[7] Lamy P Nordentoft I Birkenkamp-Demtröder K Thomsen MB Villesen P Vang S . Paired exome analysis reveals clonal evolution and potential therapeutic targets in urothelial carcinoma. Cancer Res. (2016) 76:5894–906. doi: 10.1158/0008-5472.Can-16-0436, PMID: 27488526

[8] Strandgaard T Nordentoft I Birkenkamp-Demtröder K Salminen L Prip F Rasmussen J . Field cancerization is associated with tumor development, T-cell exhaustion, and clinical outcomes in bladder cancer. Eur Urol. (2024) 85:82–92. doi: 10.1016/j.eururo.2023.07.014, PMID: 37718188

[9] Loriot Y Necchi A Park SH Garcia-Donas J Huddart R Burgess E . Erdafitinib in locally advanced or metastatic urothelial carcinoma. N Engl J Med. (2019) 381:338–48. doi: 10.1056/NEJMoa1817323, PMID: 31340094

[10] Ascione CM Napolitano F Esposito D Servetto A Belli S Santaniello A . Role of FGFR3 in bladder cancer: Treatment landscape and future challenges. Cancer Treat Rev. (2023) 115:102530. doi: 10.1016/j.ctrv.2023.102530, PMID: 36898352

[11] Noeraparast M Krajina K Pichler R Niedersüß-Beke D Shariat SF Grünwald V . FGFR3 alterations in bladder cancer: Sensitivity and resistance to targeted therapies. Cancer Commun (Lond). (2024) 44:1189–208. doi: 10.1002/cac2.12602, PMID: 39161208 PMC11483561

[12] Haas M Mayr R Sikic D Wullich B Klümper N Erben P . Clinical and genomic landscape of FGFR3 alterations across different stages of urothelial cancer. Eur Urol Open Sci. (2025) 77:1–9. doi: 10.1016/j.euros.2025.04.005, PMID: 40521274 PMC12162067

[13] Loriot Y Matsubara N Park SH Huddart RA Burgess EF Houede N . Erdafitinib or chemotherapy in advanced or metastatic urothelial carcinoma. N Engl J Med. (2023) 389:1961–71. doi: 10.1056/NEJMoa2308849, PMID: 37870920

[14] Siefker-Radtke AO Necchi A Park SH García-Donas J Huddart RA Burgess EF . Efficacy and safety of erdafitinib in patients with locally advanced or metastatic urothelial carcinoma: long-term follow-up of a phase 2 study. Lancet Oncol. (2022) 23:248–58. doi: 10.1016/s1470-2045(21)00660-4, PMID: 35030333

[15] Khaled HM Bahnassy AA Raafat AA Zekri AR Madboul MS Mokhtar NM . Clinical significance of altered nm23-H1, EGFR, RB and p53 expression in bilharzial bladder cancer. BMC Cancer. (2009) 9:32. doi: 10.1186/1471-2407-9-32, PMID: 19171060 PMC2657793

[16] Goussia AC Papoudou-Bai A Charchanti A Kitsoulis P Kanavaros P Kalef-Ezra J . Alterations of p53 and rb pathways are associated with high proliferation in bladder urothelial carcinomas. Anticancer Res. (2018) 38:3985–8. doi: 10.21873/anticanres.12685, PMID: 29970521

[17] Glaviano A Foo ASC Lam HY Yap KCH Jacot W Jones RH . PI3K/AKT/mTOR signaling transduction pathway and targeted therapies in cancer. Mol Cancer. (2023) 22:138. doi: 10.1186/s12943-023-01827-6, PMID: 37596643 PMC10436543

[18] Ho JN Byun SS Kim D Ryu H Lee S . Dasatinib induces apoptosis and autophagy by suppressing the PI3K/Akt/mTOR pathway in bladder cancer cells. Investig Clin Urol. (2024) 65:593–602. doi: 10.4111/icu.20240250, PMID: 39505519 PMC11543652

[19] Zhang L Zhang L Gao S Shi X Zuo L . Gαi3: a crucial biomarker and therapeutic target in bladder cancer. NPJ Precis Oncol. (2025) 9:181. doi: 10.1038/s41698-025-00989-4, PMID: 40514424 PMC12166043

[20] Qiu Y Wang Y Chai Z Ni D Li X Pu J . Targeting RAS phosphorylation in cancer therapy: Mechanisms and modulators. Acta Pharm Sin B. (2021) 11:3433–46. doi: 10.1016/j.apsb.2021.02.014, PMID: 34900528 PMC8642438

[21] Tong T Huang M Yan B Lin B Yu J Teng Q . Hippo signaling modulation and its biological implications in urological Malignancies. Mol Aspects Med. (2024) 98:101280. doi: 10.1016/j.mam.2024.101280, PMID: 38870717

[22] Sadhukhan P Feng M Illingworth E Sloma I Ooki A Matoso A . YAP1 induces bladder cancer progression and promotes immune evasion through IL-6/STAT3 pathway and CXCL deregulation. J Clin Invest. (2024) 135:e171164. doi: 10.1172/jci171164, PMID: 39630608 PMC11735109

[23] Cheng X Lou K Ding L Zou X Huang R Xu G . Clinical potential of the Hippo-YAP pathway in bladder cancer. Front Oncol. (2022) 12:925278. doi: 10.3389/fonc.2022.925278, PMID: 35912245 PMC9336529

[24] Maziarz M Federico A Zhao J Dujmusic L Zhao Z Monti S . Naturally occurring hotspot cancer mutations in Gα(13) promote oncogenic signaling. J Biol Chem. (2020) 295:16897–904. doi: 10.1074/jbc.AC120.014698, PMID: 33109615 PMC7864081

[25] Ghasemi H Mousavibahar SH Hashemnia M Karimi J Khodadadi I Mirzaei F . Tissue stiffness contributes to YAP activation in bladder cancer patients undergoing transurethral resection. Ann N Y Acad Sci. (2020) 1473:48–61. doi: 10.1111/nyas.14358, PMID: 32428277

[26] Ciamporcero E Daga M Pizzimenti S Roetto A Dianzani C Compagnone A . Crosstalk between Nrf2 and YAP contributes to maintaining the antioxidant potential and chemoresistance in bladder cancer. Free Radic Biol Med. (2018) 115:447–57. doi: 10.1016/j.freeradbiomed.2017.12.005, PMID: 29248722

[27] Tran L Xiao JF Agarwal N Duex JE Theodorescu D . Advances in bladder cancer biology and therapy. Nat Rev Cancer. (2021) 21:104–21. doi: 10.1038/s41568-020-00313-1, PMID: 33268841 PMC10112195

[28] Tate T Xiang T Wobker SE Zhou M Chen X Kim H . Pparg signaling controls bladder cancer subtype and immune exclusion. Nat Commun. (2021) 12:6160. doi: 10.1038/s41467-021-26421-6, PMID: 34697317 PMC8545976

[29] Plumber SA Tate T Al-Ahmadie H Chen X Choi W Basar M . Rosiglitazone and trametinib exhibit potent anti-tumor activity in a mouse model of muscle invasive bladder cancer. Nat Commun. (2024) 15:6538. doi: 10.1038/s41467-024-50678-2, PMID: 39095358 PMC11297265

[30] Rochel N Krucker C Coutos-Thévenot L Osz J Zhang R Guyon E . Recurrent activating mutations of PPARγ associated with luminal bladder tumors. Nat Commun. (2019) 10:253. doi: 10.1038/s41467-018-08157-y, PMID: 30651555 PMC6335423

[31] Jing J Wu Z Wang J Luo G Lin H Fan Y . Hedgehog signaling in tissue homeostasis, cancers, and targeted therapies. Signal Transduct Target Ther. (2023) 8:315. doi: 10.1038/s41392-023-01559-5, PMID: 37596267 PMC10439210

[32] Li J Qiu M An Y Huang J Gong C . miR-7-5p acts as a tumor suppressor in bladder cancer by regulating the hedgehog pathway factor Gli3. Biochem Biophys Res Commun. (2018) 503:2101–7. doi: 10.1016/j.bbrc.2018.07.166, PMID: 30100065

[33] Choi W Porten S Kim S Willis D Plimack ER Hoffman-Censits J . Identification of distinct basal and luminal subtypes of muscle-invasive bladder cancer with different sensitivities to frontline chemotherapy. Cancer Cell. (2014) 25:152–65. doi: 10.1016/j.ccr.2014.01.009, PMID: 24525232 PMC4011497

[34] Zhou J Zhu G Huang J Li L Du Y Gao Y . Non-canonical GLI1/2 activation by PI3K/AKT signaling in renal cell carcinoma: A novel potential therapeutic target. Cancer Lett. (2016) 370:313–23. doi: 10.1016/j.canlet.2015.11.006, PMID: 26577809

[35] Fan HX Wang S Zhao H Liu N Chen D Sun M . Sonic hedgehog signaling may promote invasion and metastasis of oral squamous cell carcinoma by activating MMP-9 and E-cadherin expression. Med Oncol. (2014) 31:41. doi: 10.1007/s12032-014-0041-5, PMID: 24915900

[36] Li X Yan X Wang Y Kaur B Han H Yu J . The Notch signaling pathway: a potential target for cancer immunotherapy. J Hematol Oncol. (2023) 16:45. doi: 10.1186/s13045-023-01439-z, PMID: 37131214 PMC10155406

[37] Robertson AG Kim J Al-Ahmadie H Bellmunt J Guo G Cherniack AD . Comprehensive molecular characterization of muscle-invasive bladder cancer. Cell. (2018) 174:1033. doi: 10.1016/j.cell.2018.07.036, PMID: 30096301 PMC6297116

[38] Van Allen EM Mouw KW Kim P Iyer G Wagle N Al-Ahmadie H . Somatic ERCC2 mutations correlate with cisplatin sensitivity in muscle-invasive urothelial carcinoma. Cancer Discov. (2014) 4:1140–53. doi: 10.1158/2159-8290.Cd-14-0623, PMID: 25096233 PMC4238969

[39] Nguyen DD Hooper WF Liu W Chu TR Geiger H Shelton JM . The interplay of mutagenesis and ecDNA shapes urothelial cancer evolution. Nature. (2024) 635:219–28. doi: 10.1038/s41586-024-07955-3, PMID: 39385020 PMC11541202

[40] Voutsadakis IA . Urothelial bladder carcinomas with high tumor mutation burden have a better prognosis and targetable molecular defects beyond immunotherapies. Curr Oncol. (2022) 29:1390–407. doi: 10.3390/curroncol29030117, PMID: 35323317 PMC8947463

[41] Alexandrov LB Kim J Haradhvala NJ Huang MN Tian Ng AW Wu Y . The repertoire of mutational signatures in human cancer. Nature. (2020) 578:94–101. doi: 10.1038/s41586-020-1943-3, PMID: 32025018 PMC7054213

[42] Petljak M Alexandrov LB Brammeld JS Price S Wedge DC Grossmann S . Characterizing mutational signatures in human cancer cell lines reveals episodic APOBEC mutagenesis. Cell. (2019) 176:1282–1294.e20. doi: 10.1016/j.cell.2019.02.012, PMID: 30849372 PMC6424819

[43] Letouzé E Shinde J Renault V Couchy G Blanc JF Tubacher E . Mutational signatures reveal the dynamic interplay of risk factors and cellular processes during liver tumorigenesis. Nat Commun. (2017) 8:1315. doi: 10.1038/s41467-017-01358-x, PMID: 29101368 PMC5670220

[44] Le DT Uram JN Wang H Bartlett BR Kemberling H Eyring AD . PD-1 blockade in tumors with mismatch-repair deficiency. N Engl J Med. (2015) 372:2509–20. doi: 10.1056/NEJMoa1500596, PMID: 26028255 PMC4481136

[45] Scimeca M Bischof J Bonfiglio R Nale E Iacovelli V Carilli M . Molecular profiling of a bladder cancer with very high tumour mutational burden. Cell Death Discov. (2024) 10:202. doi: 10.1038/s41420-024-01883-x, PMID: 38688924 PMC11061316

[46] Silva-Ferreira M Carvalho JA Salta S Henriques TS Pereira Rodrigues P Monteiro-Reis S . Diagnostic test accuracy of urinary DNA methylation-based biomarkers for the detection of primary and recurrent bladder cancer: A systematic review and meta-analysis. Eur Urol Focus. (2024) 10:922–34. doi: 10.1016/j.euf.2024.05.024, PMID: 38897871

[47] Pietrusiński M Kȩpczyński Ƚ Jȩdrzejczyk A Borkowska E Traczyk-Borszyńska M Constantinou M . Detection of bladder cancer in urine sediments by a hypermethylation panel of selected tumor suppressor genes. Cancer biomark. (2017) 18:47–59. doi: 10.3233/cbm-160673, PMID: 27814275 PMC13020615

[48] Giannopoulou AF Velentzas AD Konstantakou EG Avgeris M Katarachia SA Papandreou NC . Revisiting histone deacetylases in human tumorigenesis: the paradigm of urothelial bladder cancer. Int J Mol Sci. (2019) 20:1291. doi: 10.3390/ijms20061291, PMID: 30875794 PMC6471041

[49] Dang L White DW Gross S Bennett BD Bittinger MA Driggers EM . Cancer-associated IDH1 mutations produce 2-hydroxyglutarate. Nature. (2009) 462:739–44. doi: 10.1038/nature08617, PMID: 19935646 PMC2818760

[50] Denizio JE Dow BJ Serrano JC Ghanty U Drohat AC Kohli RM . TET-TDG active DNA demethylation at cpG and non-cpG sites. J Mol Biol. (2021) 433:166877. doi: 10.1016/j.jmb.2021.166877, PMID: 33561435 PMC8005466

[51] Wang F Li X Xie X Zhao L Chen W . UCA1, a non-protein-coding RNA up-regulated in bladder carcinoma and embryo, influencing cell growth and promoting invasion. FEBS Lett. (2008) 582:1919–27. doi: 10.1016/j.febslet.2008.05.012, PMID: 18501714

[52] Li W Jin X Zhao Y Dai J Cai Y . Long noncoding RNA GAS6-AS2 sponges microRNA-493, thereby enhancing the Malignant characteristics of breast cancer cells via upregulation of FUT4. Pathol Res Pract. (2020) 216:152772. doi: 10.1016/j.prp.2019.152772, PMID: 31839366

[53] He W Zhong G Jiang N Wang B Fan X Chen C . Long noncoding RNA BLACAT2 promotes bladder cancer-associated lymphangiogenesis and lymphatic metastasis. J Clin Invest. (2018) 128:861–75. doi: 10.1172/jci96218, PMID: 29355840 PMC5785244

[54] Chen C He W Huang J Wang B Li H Cai Q . LNMAT1 promotes lymphatic metastasis of bladder cancer via CCL2 dependent macrophage recruitment. Nat Commun. (2018) 9:3826. doi: 10.1038/s41467-018-06152-x, PMID: 30237493 PMC6148066

[55] Chen C Luo Y He W Zhao Y Kong Y Liu H . Exosomal long noncoding RNA LNMAT2 promotes lymphatic metastasis in bladder cancer. J Clin Invest. (2020) 130:404–21. doi: 10.1172/jci130892, PMID: 31593555 PMC6934220

[56] Li Q Wang H Peng H Huang Q Huyan T Huang Q . MicroRNAs: key players in bladder cancer. Mol Diagn Ther. (2019) 23:579–601. doi: 10.1007/s40291-019-00410-4, PMID: 31325035

[57] Zhang HH Huang ZX Zhong SQ Fei KL Cao YH . miR−21 inhibits autophagy and promotes Malignant development in the bladder cancer T24 cell line. Int J Oncol. (2020) 56:986–98. doi: 10.3892/ijo.2020.4984, PMID: 32319564

[58] Zhang HH Qi F Cao YH Zu XB Chen MF . Expression and clinical significance of microRNA-21, maspin and vascular endothelial growth factor-C in bladder cancer. Oncol Lett. (2015) 10:2610–6. doi: 10.3892/ol.2015.3540, PMID: 26622898 PMC4580005

[59] Zhou XU Qi L Tong S Cui YU Chen J Huang T . miR-128 downregulation promotes growth and metastasis of bladder cancer cells and involves VEGF-C upregulation. Oncol Lett. (2015) 10:3183–90. doi: 10.3892/ol.2015.3689, PMID: 26722309 PMC4665335

[60] Liu W Qi L Lv H Zu X Chen M Wang J . MiRNA-141 and miRNA-200b are closely related to invasive ability and considered as decision-making biomarkers for the extent of PLND during cystectomy. BMC Cancer. (2015) 15:92. doi: 10.1186/s12885-015-1110-7, PMID: 25884322 PMC4350852

[61] Zhang C Hu J Liu Z Deng H Xiao J Yi Z . Hsa_circ_0000520 suppresses vasculogenic mimicry formation and metastasis in bladder cancer through Lin28a/PTEN/PI3K signaling. Cell Mol Biol Lett. (2024) 29:118. doi: 10.1186/s11658-024-00627-0, PMID: 39237880 PMC11378395

[62] Qian BZ Pollard JW . Macrophage diversity enhances tumor progression and metastasis. Cell. (2010) 141:39–51. doi: 10.1016/j.cell.2010.03.014, PMID: 20371344 PMC4994190

[63] Tan WP Longo TA Inman BA . Heated intravesical chemotherapy: biology and clinical utility. Urol Clin North Am. (2020) 47:55–72. doi: 10.1016/j.ucl.2019.09.008, PMID: 31757301 PMC6917042

[64] Ghate K Amir E Kuksis M Hernandez-Barajas D Rodriguez-Romo L Booth CM . PD-L1 expression and clinical outcomes in patients with advanced urothelial carcinoma treated with checkpoint inhibitors: A meta-analysis. Cancer Treat Rev. (2019) 76:51–6. doi: 10.1016/j.ctrv.2019.05.002, PMID: 31125908

[65] Xiu W Luo J . CXCL9 secreted by tumor-associated dendritic cells up-regulates PD-L1 expression in bladder cancer cells by activating the CXCR3 signaling. BMC Immunol. (2021) 22:3. doi: 10.1186/s12865-020-00396-3, PMID: 33407095 PMC7789583

[66] Eruslanov E Neuberger M Daurkin I Perrin GQ Algood C Dahm P . Circulating and tumor-infiltrating myeloid cell subsets in patients with bladder cancer. Int J Cancer. (2012) 130:1109–19. doi: 10.1002/ijc.26123, PMID: 21480223

[67] Xu Y Zeng H Jin K Liu Z Zhu Y Xu L . Immunosuppressive tumor-associated macrophages expressing interlukin-10 conferred poor prognosis and therapeutic vulnerability in patients with muscle-invasive bladder cancer. J Immunother Cancer. (2022) 10:e003416. doi: 10.1136/jitc-2021-003416, PMID: 35338085 PMC8961180

[68] Miyake M Hori S Morizawa Y Tatsumi Y Nakai Y Anai S . CXCL1-mediated interaction of cancer cells with tumor-associated macrophages and cancer-associated fibroblasts promotes tumor progression in human bladder cancer. Neoplasia. (2016) 18:636–46. doi: 10.1016/j.neo.2016.08.002, PMID: 27690238 PMC5043399

[69] Hahn NM O’donnell MA Efstathiou JA Zahurak M Rosner GL Smith J . A phase 1 trial of durvalumab in combination with bacillus calmette-guerin (BCG) or external beam radiation therapy in patients with BCG-unresponsive non-muscle-invasive bladder cancer: the hoosier cancer research network GU16–243 ADAPT-BLADDER study. Eur Urol. (2023) 83:486–94. doi: 10.1016/j.eururo.2023.01.017, PMID: 36717286 PMC10192088

[70] Qiu S Deng L Liao X Nie L Qi F Jin K . Tumor-associated macrophages promote bladder tumor growth through PI3K/AKT signal induced by collagen. Cancer Sci. (2019) 110:2110–8. doi: 10.1111/cas.14078, PMID: 31120174 PMC6609800

[71] Berdiel-Acer M Sanz-Pamplona R Calon A Cuadras D Berenguer A Sanjuan X . Differences between CAFs and their paired NCF from adjacent colonic mucosa reveal functional heterogeneity of CAFs, providing prognostic information. Mol Oncol. (2014) 8:1290–305. doi: 10.1016/j.molonc.2014.04.006, PMID: 24839936 PMC5528579

[72] Cheng HW Chen YF Wong JM Weng CW Chen HY Yu SL . Cancer cells increase endothelial cell tube formation and survival by activating the PI3K/Akt signalling pathway. J Exp Clin Cancer Res. (2017) 36:27. doi: 10.1186/s13046-017-0495-3, PMID: 28173828 PMC5296960

[73] Mazzola CR Chin J . Targeting the VEGF pathway in metastatic bladder cancer. Expert Opin Investig Drugs. (2015) 24:913–27. doi: 10.1517/13543784.2015.1041588, PMID: 26098435

[74] Lopez-Beltran A Cimadamore A Blanca A Massari F Vau N Scarpelli M . Immune checkpoint inhibitors for the treatment of bladder cancer. Cancers (Basel). (2021) 13:131. doi: 10.3390/cancers13010131, PMID: 33401585 PMC7795541

[75] Chen DS Mellman I . Oncology meets immunology: the cancer-immunity cycle. Immunity. (2013) 39:1–10. doi: 10.1016/j.immuni.2013.07.012, PMID: 23890059

[76] Rosenberg JE Hoffman-Censits J Powles T van der Heijden MS Balar AV Necchi A . Atezolizumab in patients with locally advanced and metastatic urothelial carcinoma who have progressed following treatment with platinum-based chemotherapy: a single-arm, multicentre, phase 2 trial. Lancet. (2016) 387:1909–20. doi: 10.1016/s0140-6736(16)00561-4, PMID: 26952546 PMC5480242

[77] Vander Heiden MG Cantley LC Thompson CB . Understanding the Warburg effect: the metabolic requirements of cell proliferation. Science. (2009) 324:1029–33. doi: 10.1126/science.1160809, PMID: 19460998 PMC2849637

[78] Christofk HR Vander Heiden MG Harris MH Ramanathan A Gerszten RE Wei R . The M2 splice isoform of pyruvate kinase is important for cancer metabolism and tumour growth. Nature. (2008) 452:230–3. doi: 10.1038/nature06734, PMID: 18337823

[79] Zhang H Li Y Huang J Shen L Xiong Y . Precise targeting of lipid metabolism in the era of immuno-oncology and the latest advances in nano-based drug delivery systems for cancer therapy. Acta Pharm Sin B. (2024) 14:4717–37. doi: 10.1016/j.apsb.2024.07.021, PMID: 39664426 PMC11628863

[80] Hao S Shen L Liu P Yong Q Wang Y Zheng X . Development of a prognostic model for muscle-invasive bladder cancer using glutamine metabolism. Comput Biol Med. (2024) 171:108223. doi: 10.1016/j.compbiomed.2024.108223, PMID: 38430744

[81] Attanasio G Failla M Poidomani S Buzzanca T Salzano S Zizzo M . Histological and immunohistochemical approaches to molecular subtyping in muscle-invasive bladder cancer. Front Oncol. (2025) 15:1546160. doi: 10.3389/fonc.2025.1546160, PMID: 40735045 PMC12303812

[82] Groeneveld CS Sanchez-Quiles V Dufour F Shi M Dingli F Nicolle R . Proteogenomic characterization of bladder cancer reveals sensitivity to apoptosis induced by tumor necrosis factor-related apoptosis-inducing ligand in FGFR3-mutated tumors. Eur Urol. (2024) 85:483–94. doi: 10.1016/j.eururo.2023.05.037, PMID: 37380559

[83] Inamura K . Bladder cancer: new insights into its molecular pathology. Cancers (Basel). (2018) 10:100. doi: 10.3390/cancers10040100, PMID: 29614760 PMC5923355

[84] Cotillas EA Bernardo C Veerla S Liedberg F Sjödahl G Eriksson P . A versatile and upgraded version of the lundTax classification algorithm applied to independent cohorts. J Mol Diagn. (2024) 26:1081–101. doi: 10.1016/j.jmoldx.2024.08.005, PMID: 39326668

[85] Siefker-Radtke AO Matsubara N Park SH Huddart RA Burgess EF Özgüroğlu M . Erdafitinib versus pembrolizumab in pretreated patients with advanced or metastatic urothelial cancer with select FGFR alterations: cohort 2 of the randomized phase III THOR trial. Ann Oncol. (2024) 35:107–17. doi: 10.1016/j.annonc.2023.10.003, PMID: 37871702

[86] Peng J Sridhar S Siefker-Radtke AO Selvarajah S Jiang DM . Targeting the FGFR pathway in urothelial carcinoma: the future is now. Curr Treat Options Oncol. (2022) 23:1269–87. doi: 10.1007/s11864-022-01009-4, PMID: 35962938

[87] Necchi A Pouessel D Leibowitz R Gupta S Fléchon A García-Donas J . Pemigatinib for metastatic or surgically unresectable urothelial carcinoma with FGF/FGFR genomic alterations: final results from FIGHT-201. Ann Oncol. (2024) 35:200–10. doi: 10.1016/j.annonc.2023.10.794, PMID: 37956738

[88] Pulido M Roubaud G Cazeau AL Mahammedi H Vedrine L Joly F . Safety and efficacy of temsirolimus as second line treatment for patients with recurrent bladder cancer. BMC Cancer. (2018) 18:194. doi: 10.1186/s12885-018-4059-5, PMID: 29454321 PMC5816357

[89] Gerullis H Eimer C Ecke TH Georgas E Freitas C Kastenholz S . A phase II trial of temsirolimus in second-line metastatic urothelial cancer. Med Oncol. (2012) 29:2870–6. doi: 10.1007/s12032-012-0216-x, PMID: 22447503

[90] Bellmunt J De Wit R Vaughn DJ Fradet Y Lee JL Fong L . Pembrolizumab as second-line therapy for advanced urothelial carcinoma. N Engl J Med. (2017) 376:1015–26. doi: 10.1056/NEJMoa1613683, PMID: 28212060 PMC5635424

[91] Hahn NM Stadler WM Zon RT Waterhouse D Picus J Nattam S . Phase II trial of cisplatin, gemcitabine, and bevacizumab as first-line therapy for metastatic urothelial carcinoma: Hoosier Oncology Group GU 04-75. J Clin Oncol. (2011) 29:1525–30. doi: 10.1200/jco.2010.31.6067, PMID: 21422406

[92] Von Der Maase H Hansen SW Roberts JT Dogliotti L Oliver T Moore MJ . Gemcitabine and cisplatin versus methotrexate, vinblastine, doxorubicin, and cisplatin in advanced or metastatic bladder cancer: results of a large, randomized, multinational, multicenter, phase III study. J Clin Oncol. (2000) 18:3068–77. doi: 10.1200/jco.2000.18.17.3068, PMID: 11001674

[93] Fleischmann A Rotzer D Seiler R Studer UE Thalmann GN . Her2 amplification is significantly more frequent in lymph node metastases from urothelial bladder cancer than in the primary tumours. Eur Urol. (2011) 60:350–7. doi: 10.1016/j.eururo.2011.05.035, PMID: 21640482

[94] Sheng X Yan X Wang L Shi Y Yao X Luo H . Open-label, multicenter, phase II study of RC48-ADC, a HER2-targeting antibody-drug conjugate, in patients with locally advanced or metastatic urothelial carcinoma. Clin Cancer Res. (2021) 27:43–51. doi: 10.1158/1078-0432.Ccr-20-2488, PMID: 33109737

[95] Kumbham S Md Mahabubur Rahman K Foster BA You Y . A comprehensive review of current approaches in bladder cancer treatment. ACS Pharmacol Transl Sci. (2025) 8:286–307. doi: 10.1021/acsptsci.4c00663, PMID: 39974639 PMC11833730

[96] Zhang Y Wang X Li J Zhang P Hu H . Global clinical trial landscape and therapeutic trends in bladder cancer: a systematic analysis. Int J Surg. (2025) 111:6449–52. doi: 10.1097/js9.0000000000002662, PMID: 40503775 PMC12430826

[97] Santoni M Myint ZW Büttner T Takeshita H Okada Y Lam ET . Real-world effectiveness of pembrolizumab as first-line therapy for cisplatin-ineligible patients with advanced urothelial carcinoma: the ARON-2 study. Cancer Immunol Immunother. (2023) 72:2961–70. doi: 10.1007/s00262-023-03469-5, PMID: 37248424 PMC10991859

[98] Balar AV Kamat AM Kulkarni GS Uchio EM Boormans JL Roumiguié M . Pembrolizumab monotherapy for the treatment of high-risk non-muscle-invasive bladder cancer unresponsive to BCG (KEYNOTE-057): an open-label, single-arm, multicentre, phase 2 study. Lancet Oncol. (2021) 22:919–30. doi: 10.1016/s1470-2045(21)00147-9, PMID: 34051177

[99] Oyler HJ Bruton LG Maher AJ Yu DA Shely NW Wakefield MR . Cytokine therapy in bladder cancer: mechanisms, efficacy, and future prospects. Curr Issues Mol Biol. (2025) 47:278. doi: 10.3390/cimb47040278, PMID: 40699676 PMC12025466

[100] Chu C Pietzak E . Immune mechanisms and molecular therapeutic strategies to enhance immunotherapy in non-muscle invasive bladder cancer: Invited review for special issue “Seminar: Treatment Advances and Molecular Biology Insights in Urothelial Carcinoma. Urol Oncol. (2023) 41:398–409. doi: 10.1016/j.urolonc.2022.05.013, PMID: 35811207 PMC10167944

[101] Rosenberg JE Galsky MD Powles T Petrylak DP Bellmunt J Loriot Y . Atezolizumab monotherapy for metastatic urothelial carcinoma: final analysis from the phase II IMvigor210 trial. ESMO Open. (2024) 9:103972. doi: 10.1016/j.esmoop.2024.103972, PMID: 39642637 PMC11667038

[102] Powles T Park SH Voog E Caserta C Valderrama BP Gurney H . Avelumab maintenance therapy for advanced or metastatic urothelial carcinoma. N Engl J Med. (2020) 383:1218–30. doi: 10.1056/NEJMoa2002788, PMID: 32945632

[103] Grivas P Kopyltsov E Su PJ Parnis FX Park SH Yamamoto Y . Patient-reported outcomes from JAVELIN bladder 100: avelumab first-line maintenance plus best supportive care versus best supportive care alone for advanced urothelial carcinoma. Eur Urol. (2023) 83:320–8. doi: 10.1016/j.eururo.2022.04.016, PMID: 35654659

[104] Han J Gu X Li Y Wu Q . Mechanisms of BCG in the treatment of bladder cancer-current understanding and the prospect. BioMed Pharmacother. (2020) 129:110393. doi: 10.1016/j.biopha.2020.110393, PMID: 32559616

[105] Hannouneh ZA Hijazi A Alsaleem AA Hami S Kheyrbek N Tanous F . Novel immunotherapeutic options for BCG-unresponsive high-risk non-muscle-invasive bladder cancer. Cancer Med. (2023) 12:21944–68. doi: 10.1002/cam4.6768, PMID: 38037752 PMC10757155

[106] Powles T Csőszi T Özgüroğlu M Matsubara N Géczi L Cheng SY . Pembrolizumab alone or combined with chemotherapy versus chemotherapy as first-line therapy for advanced urothelial carcinoma (KEYNOTE-361): a randomised, open-label, phase 3 trial. Lancet Oncol. (2021) 22:931–45. doi: 10.1016/s1470-2045(21)00152-2, PMID: 34051178

[107] Grande E Arranz J De Santis M Bamias A Kikuchi E Del Muro XG . Atezolizumab plus chemotherapy versus placebo plus chemotherapy in untreated locally advanced or metastatic urothelial carcinoma (IMvigor130): final overall survival analysis results from a randomised, controlled, phase 3 study. Lancet Oncol. (2024) 25:29–45. doi: 10.1016/s1470-2045(23)00540-5, PMID: 38101433

[108] Basile G Bandini M Gibb EA Ross JS Raggi D Marandino L . Neoadjuvant pembrolizumab and radical cystectomy in patients with muscle-invasive urothelial bladder cancer: 3-year median follow-up update of PURE-01 trial. Clin Cancer Res. (2022) 28:5107–14. doi: 10.1158/1078-0432.Ccr-22-2158, PMID: 36190522

[109] Boorjian SA Alemozaffar M Konety BR Shore ND Gomella LG Kamat AM . Intravesical nadofaragene firadenovec gene therapy for BCG-unresponsive non-muscle-invasive bladder cancer: a single-arm, open-label, repeat-dose clinical trial. Lancet Oncol. (2021) 22:107–17. doi: 10.1016/s1470-2045(20)30540-4, PMID: 33253641 PMC7988888

[110] Ding S Liu J Han X Tang M . CRISPR/cas9-mediated genome editing in cancer therapy. Int J Mol Sci. (2023) 24:16325. doi: 10.3390/ijms242216325, PMID: 38003514 PMC10671490

[111] Tse RT Zhao H Wong CY Cheng CK Kong AW Peng Q . Urinary cell-free DNA in bladder cancer detection. Diagn (Basel). (2021) 11:306. doi: 10.3390/diagnostics11020306, PMID: 33672869 PMC7918217

[112] Shindo T Shimizu T Nojima M Niinuma T Maruyama R Kitajima H . Evaluation of urinary DNA methylation as a marker for recurrent bladder cancer: A 2-center prospective study. Urology. (2018) 113:71–8. doi: 10.1016/j.urology.2017.11.025, PMID: 29196070

[113] Zang J Zhang R Jin D Xie F Shahatiaili A Wu G . Integrated longitudinal circulating tumor DNA profiling predicts immunotherapy response of metastatic urothelial carcinoma in the POLARIS-03 trial. J Pathol. (2023) 261:198–209. doi: 10.1002/path.6166, PMID: 37584165

[114] Sfakianos JP Basu A Laliotis G Cumarasamy S Rich JM Kommalapati A . Association of tumor-informed circulating tumor DNA detectability before and after radical cystectomy with disease-free survival in patients with bladder cancer. Eur Urol Oncol. (2025) 8:306–14. doi: 10.1016/j.euo.2024.07.001, PMID: 39013741

[115] Ben-David R Tillu N Cumarasamy S Alerasool P Rich JM Kaufmann B . Longitudinal tumor-informed circulating tumor DNA status predicts disease upstaging and poor prognosis for patients undergoing radical cystectomy. Eur Urol Oncol. (2024) 7:1105–12. doi: 10.1016/j.euo.2024.03.002, PMID: 38521660

[116] Palugan L Cerea M Cirilli M Moutaharrik S Maroni A Zema L . Intravesical drug delivery approaches for improved therapy of urinary bladder diseases. Int J Pharm X. (2021) 3:100100. doi: 10.1016/j.ijpx.2021.100100, PMID: 34765967 PMC8569723

[117] Yang FK Tian C Zhou LX Guan TY Chen GL Zheng YY . The value of urinary exosomal microRNA-21 in the early diagnosis and prognosis of bladder cancer. Kaohsiung J Med Sci. (2024) 40:660–70. doi: 10.1002/kjm2.12845, PMID: 38801488 PMC11895653

[118] Huang XL Zhang H Yang XY Dong XY Xie XY Yin HB . Activation of a c-Jun N-terminal kinase-mediated autophagy pathway attenuates the anticancer activity of gemcitabine in human bladder cancer cells. Anticancer Drugs. (2017) 28:596–602. doi: 10.1097/cad.0000000000000499, PMID: 28430744

[119] Dreyer T Brandt S Fabrin K Azawi N Vásquez JL Ernst A . Use of the xpert bladder cancer monitor urinary biomarker test for guiding cystoscopy in high-grade non-muscle-invasive bladder cancer: results from the randomized controlled daBlaCa-15 trial. Eur Urol. (2025) 88:23–30. doi: 10.1016/j.eururo.2025.03.018, PMID: 40280776

[120] Christensen E Birkenkamp-Demtröder K Sethi H Shchegrova S Salari R Nordentoft I . Early detection of metastatic relapse and monitoring of therapeutic efficacy by ultra-deep sequencing of plasma cell-free DNA in patients with urothelial bladder carcinoma. J Clin Oncol. (2019) 37:1547–57. doi: 10.1200/jco.18.02052, PMID: 31059311

[121] Smith SC Baras AS Dancik G Ru Y Ding KF Moskaluk CA . A 20-gene model for molecular nodal staging of bladder cancer: development and prospective assessment. Lancet Oncol. (2011) 12:137–43. doi: 10.1016/s1470-2045(10)70296-5, PMID: 21256081 PMC3613042

[122] Seiler R Lam LL Erho N Takhar M Mitra AP Buerki C . Prediction of lymph node metastasis in patients with bladder cancer using whole transcriptome gene expression signatures. J Urol. (2016) 196:1036–41. doi: 10.1016/j.juro.2016.04.061, PMID: 27105761

[123] Thompson D Lawrentschuk N Bolton D . New approaches to targeting epigenetic regulation in bladder cancer. Cancers (Basel). (2023) 15:1856. doi: 10.3390/cancers15061856, PMID: 36980741 PMC10046617

[124] Jäger T Tschirdewahn S Vom Dorp F Piechotta G Rübben H Szarvas T . Siliconchiptechnology-based MMP-7 analysis in urine: an option for preoperative identification of lymph node metastasis in bladder cancer. Urol A. (2013) 52:853–8. doi: 10.1007/s00120-012-3110-4, PMID: 23358831

[125] Wu S Zheng J Li Y Yu H Shi S Xie W . A radiomics nomogram for the preoperative prediction of lymph node metastasis in bladder cancer. Clin Cancer Res. (2017) 23:6904–11. doi: 10.1158/1078-0432.Ccr-17-1510, PMID: 28874414

[126] Wu S Zheng J Li Y Wu Z Shi S Huang M . Development and validation of an MRI-based radiomics signature for the preoperative prediction of lymph node metastasis in bladder cancer. EBioMedicine. (2018) 34:76–84. doi: 10.1016/j.ebiom.2018.07.029, PMID: 30078735 PMC6116473

[127] Babjuk M Böhle A Burger M Capoun O Cohen D Compérat EM . EAU guidelines on non-muscle-invasive urothelial carcinoma of the bladder: update 2016. Eur Urol. (2017) 71:447–61. doi: 10.1016/j.eururo.2016.05.041, PMID: 27324428

[128] Ahmadi H Duddalwar V Daneshmand S . Diagnosis and staging of bladder cancer. Hematol Oncol Clin North Am. (2021) 35:531–41. doi: 10.1016/j.hoc.2021.02.004, PMID: 33958149

[129] Bošković M Roje B Chung FF Gelemanović A Cahais V Cuenin C . DNA methylome changes of muscle- and neuronal-related processes precede bladder cancer invasiveness. Cancers (Basel). (2022) 14:487. doi: 10.3390/cancers14030487, PMID: 35158756 PMC8833512

[130] Grivas P Garralda E Meric-Bernstam F Mellinghoff IK Goyal L Harding JJ . Evaluating debio 1347 in patients with FGFR fusion-positive advanced solid tumors from the FUZE multicenter, open-label, phase II basket trial. Clin Cancer Res. (2024) 30:4572–83. doi: 10.1158/1078-0432.Ccr-24-0012, PMID: 38771739 PMC11707795

